# Cognitive arbitration between candidate dimensions of psychopathology

**DOI:** 10.1038/s41380-025-03297-2

**Published:** 2025-10-15

**Authors:** Celine A. Fox, Vanessa Teckentrup, Kelly R. Donegan, Tricia XF Seow, Christopher SY Benwell, Brenden Tervo-Clemmens, Claire M. Gillan

**Affiliations:** 1 School of Psychology, Trinity College Dublin, Dublin, Ireland; 2 Trinity College Institute of Neuroscience, Trinity College Dublin, Dublin, Ireland; 3 Functional Imaging Laboratory, University College London, London, UK; 4 Max Planck UCL Centre for Computational Psychiatry and Ageing Research, University College London, London, UK; 5 Division of Psychology, School of Humanities, Social Sciences and Law, University of Dundee, Dundee, UK; 6 Department of Psychiatry & Behavioral Sciences, University of Minnesota, Minneapolis, MN USA

**Keywords:** Neuroscience, Psychiatric disorders, Psychology

## Abstract

As an alternative to the Diagnostic and Statistical Manual of Mental Disorders, transdiagnostic approaches that identify latent dimensions of psychopathology through factor analysis have gained prominence in recent years. A key critique of these approaches, however, is that they are performed at the level of symptoms only. This begs the question: are these dimensions truly more valid predictors of external outcomes than existing alternatives? Are there other ways, that are more data-driven, which can allow us to refine our definitions of clinical phenotypes? We tested this idea empirically, conducting a large-scale meta-scientific comparison of thousands of competing factor solutions that allowed us to determine if the latent structure underlying the covariation of psychiatric symptoms has robust and specific cognitive correlates. In nine independent datasets, comprising *N* = 7565 individuals including patients about to start mental health treatment, healthy individuals, paid and unpaid participants, a broad set of age ranges and cognitive task variants that measured model-based planning and metacognition, we found that factors with the best fit to cognition were those derived from a first-order factor analysis on the maximal number of theoretically informed self-report symptoms available. These factors (‘Compulsivity and Intrusive Thought’ and ‘Anxious-depression’) performed better than thousands of engineered alternatives and performed twice as well as traditional questionnaire total scores. Crucially, this unsupervised approached based on symptom correlation only performed on-par with a partial least squares analysis, a supervised approach to deriving factors based on cognition. These results provide evidence that unsupervised factor analysis of psychiatric symptoms is a viable method for rethinking how we define mental health and illness, affording clear opportunities for enhancing our understanding of specific underlying mechanistic processes.

## Introduction

Dimensional and transdiagnostic approaches to psychiatric classification have gained traction over the last decade [1, 2], positioned as an alternative to the Diagnostic and Statistical Manual of Mental Disorders (DSM) [3]. The goal of these new efforts are to resolve long-standing issues including the problem of symptom heterogeneity within DSM diagnostic categories [4, 5], the overlap in symptoms across different diagnoses [6], the high rates of co-morbidity [7, 8] and the reduction of continuous clinical information into binary categories [9, 10]. These challenges compromise both the validity and reliability of clinical phenotypes and this has, in turn, translated into a lack of progress in developing a mechanistic understanding of mental health [11–13]. As a result, differences in cognitive functioning associated with clinical diagnoses are destined to be small and disorder non-specific [14, 15], as are genetic [16] and brain-behaviour associations [17–19]. To achieve progress in our mechanistic understanding of psychiatric conditions, there is a growing need for an empirically derived, valid and reliable, set of transdiagnostic dimensional clinical phenotypes. Without this, a low ceiling is placed on the size and specificity of our scientific observations that cannot be addressed by even the most exciting advances in neuroimaging techniques [20], computational modelling [21] or artificial intelligence [22].

Although transdiagnostic, dimensional approaches to psychopathology have many proponents [2, 23] and are central to major funding initiatives, such as the Research Domain Criteria (RDoC) [24], there is not yet a consensus around how candidate dimensions should be identified and validated. One increasingly popular approach is to derive dimensions by examining the natural covariation of symptoms between-subjects using factor analysis, as is the foundation of the Hierarchical Taxonomy of Psychopathology (HiTOP) [25] and Computational Factor modelling approaches [26]. A key conceptual critique of this approach, however, is that it operates at the symptom level only [27]. This is a problem because as symptoms that hang together do not necessarily have a shared aetiology [28, 29]. Rather, a single dimension of related symptoms across DSM disorders can be reached via multiple mechanistic pathways [28]. Symptoms can cause one-another [30], the same symptoms can be arrived at from different causes and conversely, a single underlying cause can produce different symptoms in different individuals for example depending on early childhood experiences [31]. For these reasons, it is not certain if a covariation-based framework is well positioned to advance beyond DSM and deliver clinical phenotypes that have a firm mechanistic grounding.

Emerging evidence has shown promise, however. Transdiagnostic dimensions derived from factor analysis of a large set of individual symptoms outperform traditional questionnaire scores in their association with some cognitive-computational processes. For example, model-based planning, the tendency to use prospective mental maps to guide behaviour [32], has a stronger and more specific association to a transdiagnostic dimension ‘Compulsivity and Intrusive Thought’ than traditional disorder categories or self-report questionnaires [33–36]. Metacognitive bias - the confidence one has in their own performance [37] – tends to be decreased in a non-specific way across many DSM disorder categories and questionnaires [38], but has a highly specific, bi-directional associations with two different transdiagnostic dimensions. Individuals high in ‘Anxious-depression’ are underconfident, whereas those with ‘Compulsivity and Intrusive Thought’ express overconfidence [39–44].

These examples suggest that transdiagnostic clinical phenotypes derived from factor analysis of symptoms may offer an advance over traditional diagnoses and related self-report instruments [26]. However, a major outstanding question concerns whether factor analysis is really optimised for this task, particularly given the limitations described above. Clinical dimensions that map even more closely to cognition may exist, but may not correspond to the most substantial axes of variation in a given questionnaire set and so would not be selected in factor analysis. Moreover, factor solutions are fundamentally constrained by the specific dataset at hand; they change depending on the inclusion or omission of symptoms or diagnoses [45]. In addition, there are multiple degrees of analytic freedom [29], for example regarding the number of factors retained [46], the inclusion of a higher-order general factor [47], and whether dimensions of psychopathology should be orthogonal or partially correlated [48]. There is currently no consensus on these issues and without this, there exists a large, possibly infinite, set of alternative and well-fitting models [49]. In sum, these models are designed to find the underlying latent structure of the symptoms provided, and it is unclear if the best solution to this problem corresponds to the most mechanistically-valid dimensions of psychopathology.

This paper aimed to answer this question by using data from over 7000 individuals from nine independent datasets [33–36, 39, 40, 42, 43, 50], with different versions of cognitive tasks measuring model-based planning and metacognition, various age ranges, data-collection methods, and representing a spread of general, crowd-sourced and clinical populations. To generate an analytic consensus on the structure of psychopathology, we generated thousands of competing factor solutions, spanning variations in the number of factors retained, a multiverse of expanded and contracted questionnaire sets, and examining bifactor, orthogonal and oblique rotations and tested for the maximal association with the cognitive-computational capacity of interest. We benchmarked performance against a baseline of established transdiagnostic dimensions (i.e., ‘Anxious-depression’ and ‘Compulsivity and Intrusive Thought’) [34], which were derived from a 3-factor solution, an oblique rotation, and based on the full set of items. Finally, we compared the mechanistic validity and identity of factors derived from the covariance of symptoms alone versus a fully supervised method (partial least squares ‘PLS’ regression) that uses cognitive information to inform the derivation of factors.

## Results

### Generating a transdiagnostic factor multiverse

**Fig. 1:**
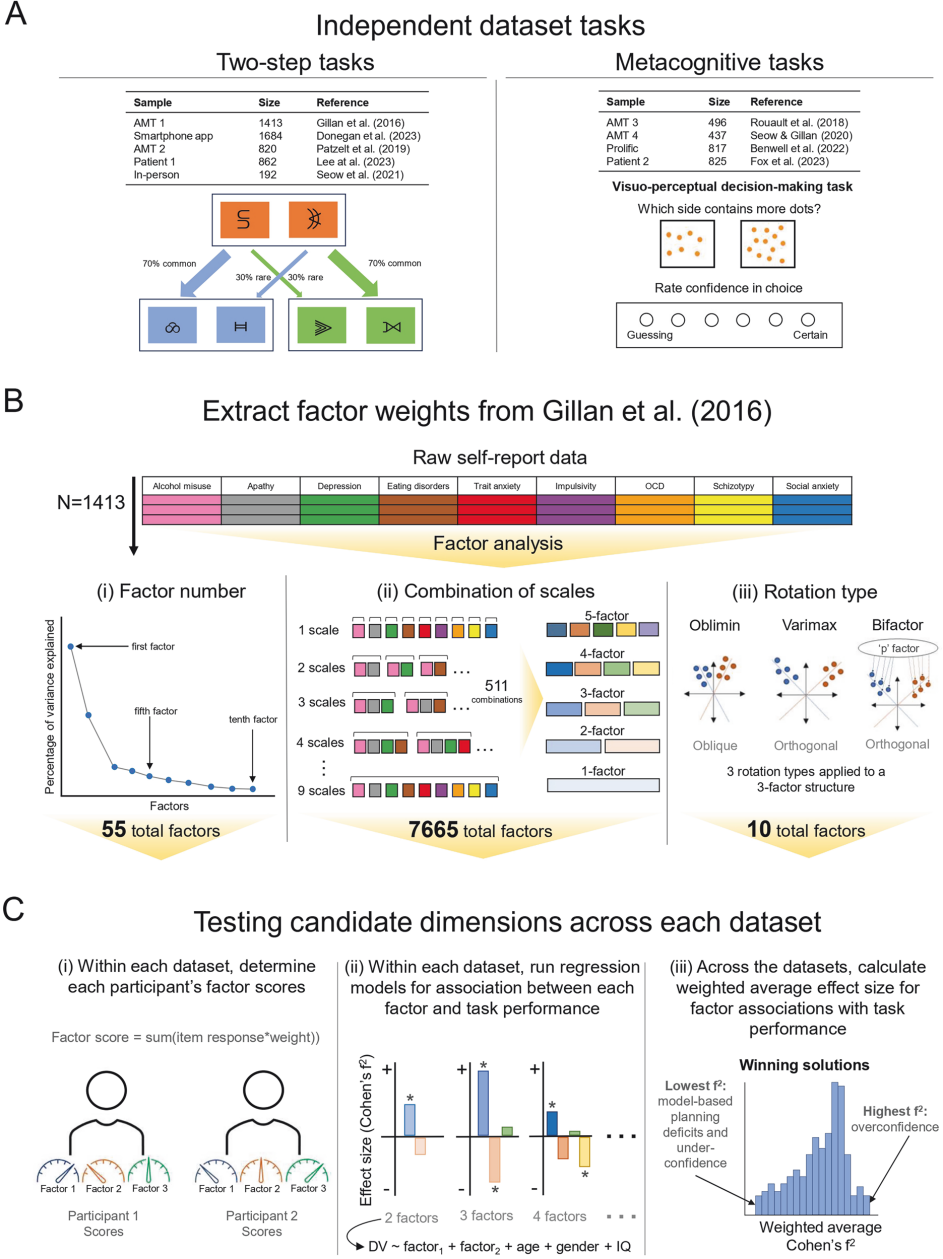
General Methodology. AMT Amazon’s Mechanical Turk. The four AMT and two patient datasets are numerically labelled to help distinguish samples (e.g., AMT 1 = Gillan et al. [34] dataset (*N =* 1413)). **A** Five datasets included alternative versions of the two-step task to assess model-based planning abilities. Four datasets included metacognitive tasks: Two included alternative versions of a visuo-perceptual decision-making task, one used both visuo-perceptual and knowledge decision-making tasks, and one dataset used a predictive inference task. **B** Factor weights were extracted from a discovery dataset [34], following separate manipulations of (i) the number of factors retained (ranging from a single- to 10-factor structure, resulting in 55 total factors), (ii) the combinations of scales (ranging from a single questionnaire to all nine questionnaires considered, with each combination subsequently analysed as a single- to 5-factor structure, resulting in 7665 total factors), and (iii) the rotation type (oblique ‘oblimin’, orthogonal ‘varimax’ or ‘bifactor’ rotations, each as a three-factor structure (in addition to a hierarchical factor for the bifactor rotation), resulting in 10 total factor solutions). **C** (i) The performance of each dimension was assessed by examining how within dataset factor scores (ii) were associated with model-based planning/metacognitive bias, controlling for age, gender and IQ/education, using linear regression analyses to extract the effect size (Cohen’s f^2^ value of each factor). (iii) The weighted average Cohen’s f^2^ values across datasets was used to determine which factor had the largest ‘winning’ effect size for predicting individual differences in model-based planning (lowest Cohen’s f^2^ value), overconfidence (highest Cohen’s f^2^ value), and underconfidence (lowest Cohen’s f^2^ value) respectively.

In a factor discovery dataset of *N* = 1413 individuals gathered from Amazon’s Mechanical Turk (AMT) [34], we generated thousands of possible factor solutions based on a core dataset of 209 questionnaire items. These measured symptoms of obsessive compulsive disorder (OCD), eating disorders, impulsivity, schizotypy, apathy, alcohol addiction, depression, trait anxiety and social anxiety [34]. Using this set, we examined how variations in the number of factors retained per model (1- to 10-factor solutions, *n =* 55 factors; Fig. 1B(i)) affects the association between resulting dimensions and model-based planning and metacognition. We then tested the impact of the size and composition of the questionnaire set selected for analysis on the resulting factor solutions. To do this, we generated every possible combination of the nine self-report clinical scales (*N* = 511) and factor-analysed the item-level responses, specifying an oblique (‘oblimin’) rotation and considering solutions retaining up to 5 factors (15 possible factors per model), resulting in 7665 candidate dimensions (Fig. 1B(ii)). We followed this with a comparison of factors derived from oblique, orthogonal rotations and a bifactor model (Fig. 1B(iii), *n =* 10 factors). Mechanistic validity was defined as the magnitude of the effect size of a given dimensions in predicting a given cognitive capacity (i.e., positive metacognitive bias, negative metacognitive bias and model-based planning), controlling for age, gender and education/IQ. This was calculated across several datasets and summarised in a weighted average.

### Comparing transdiagnostic dimensions to questionnaire total scores

**Fig. 2:**
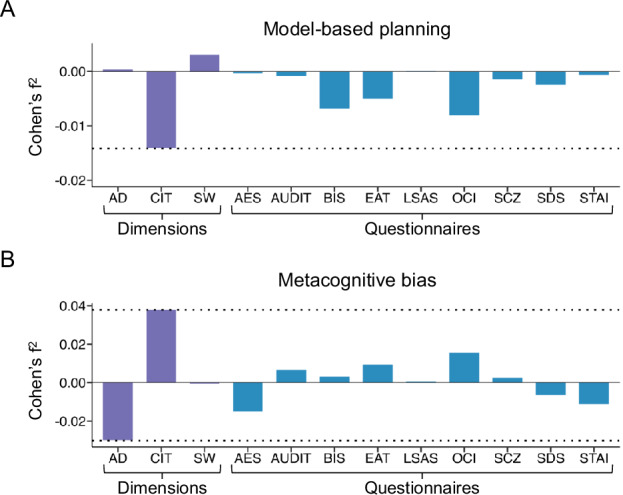
Weighted average effect sizes of the association between cognitive test data and total scores on clinical questionnaires vs. transdiagnostic dimensions. AD Anxious-depression, CIT Compulsivity and Intrusive Thought, SW Social Withdrawal, AES Apathy Evaluation Scale, AUDIT Alcohol Use Disorders Identification Test, BIS Barratt Impulsiveness Scale, EAT Eating Attitudes Test, LSAS Liebowitz Social Anxiety Scale, OCI Obsessive-Compulsive Inventory-Revised, SCZ Short Scales for Measuring Schizotypy, SDS Zung Self-Rating Depression Scale, STAI State Trait Anxiety Inventory. **A** Weighted effect sizes of dimensions and questionnaires from linear regression analyses predicting model-based planning, averaged across 5 datasets (*N =* 4990). **B** Weighted effect sizes of dimensions and questionnaires from linear regression analyses predicting metacognitive bias, averaged across 4 datasets (*N =* 2575).

As a benchmarking step, we calculated the weighted average effect sizes for associations with model-based planning and metacognitive bias and (i) total scores across the nine clinical questionnaire vs (ii) the established transdiagnostic dimensions (Fig. 2). We conducted separate regression analyses per cognitive task within each dataset, with the questionnaires/dimensions as an independent variable, along with age, gender and education/IQ. Regression analyses with clinical questionnaires included each questionnaire in a separate model (e.g., metacognitive bias ~ depression scale score + age + gender + IQ), while regression analyses for dimensions included the three dimensions in the same model (e.g., metacognitive bias ~ Anxious-depression + Compulsivity and Intrusive Thought + Social Withdrawal + age + gender + IQ). Examining the weighted average effect sizes (Cohen’s f^2^) across datasets for each questionnaire and dimension, transdiagnostic dimensions outperformed clinical questionnaires across all cognitive facets (Fig. 2). For reductions in model-based planning, ‘Compulsivity and Intrusive Thought’ was the top performing factor (Cohen’s f^2^ = −0.014), performing better than all the clinical questionnaires (Fig. 2A). Similar for metacognitive bias, ‘Compulsivity and Intrusive Thought’ had the largest positive effect size for individual differences in overconfidence (Cohen’s f^2^ = 0.038), and ‘Anxious-depression’ had the largest negative effect size for individual differences in underconfidence (Cohen’s f^2^ = −0.030) (Fig. 2B). The effect sizes of ‘Compulsivity and Intrusive Thought’ and ‘Anxious-depression’ for metacognitive bias were twice as large as the effect sizes of the next best performing questionnaires (the Obsessive-Compulsive Inventory-Revised for overconfidence with a Cohen’s f^2^ = 0.016, and the Apathy Evaluation Scale with a Cohen’s f^2^ = −0.015 for underconfidence) (Fig. 2B). The 3-factor solution had a root mean square error of approximation (RMSEA) of 0.04 and a Turker-Lewis Index (TLI) of 0.53 and is taken as the benchmark for subsequent analyses.

### Variation of the number of factors selected for retention

**Fig. 3:**
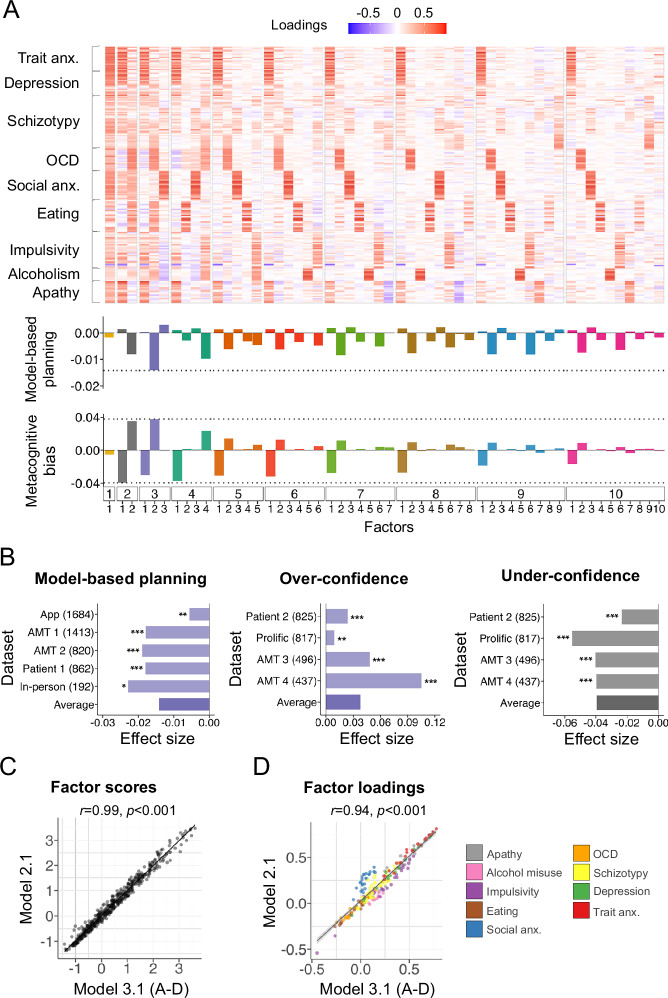
Variation of the number of factors selected for retention. AMT Amazon’s Mechanical Turk. A-D Anxious-depression, OCD Obsessive compulsive disorder, Social anx Social anxiety, Trait anx Trait anxiety, r Pearson correlation coefficient, p p-value. **A** Fifty-five factors were generated from solutions retaining 1-factor, 2-factors, …, 10-factors. Heatmap indicates the loading of individual items onto each resulting factor (top panel). Weighted effect sizes (f^2^) from 10 linear regression analyses predicting model-based planning, averaged across 5 datasets (*N =* 4990) (middle panel) and metacognition in 4 datasets (*N =* 2575) (bottom panel). Factor loadings and effect sizes are aligned along the same x-axis. **B** Top-performing factors’ effect sizes within each dataset and the weighted averaged effects across datasets. The top-performing factors had a statistically significant effects on model-based planning overconfidence and underconfidence within each dataset (all *p* < 0.05). *p < 0.05, **p < 0.01, ***p < 0.001. The top performing factor for model-based planning and overconfidence was extracted from the 3-factor model (in purple) and the top performing factor for underconfidence came from the 2-factor model (in grey). **C** Correlation between scores on the first factor in a 3-factor solution (model 3.1 i.e., ‘Anxious-depression’) and the first factor from a 2-factor solution (model 2.1) in the discovery dataset. **D** Correlation between factor loadings of ‘Anxious-depression’ and model 2.1 in the discovery dataset.

Factors that do not represent a significant axis of variation (based on our selection criteria, see below) in a questionnaire set may not be selected for retention; but they may nonetheless constitute an important and mechanistically homogenous dimension of psychopathology. To test this (Fig. 1B(i)), we dispensed with principled approaches to determine the ideal number of factors to retain and instead generated all possible factors up to a maximum 10 factor solution. This gave a total of 55 factors, with the first from a 1-factor solution, the following 2 from a 2-factor solution, all the way to a 10-factor model (Fig. 2A). For model-based planning, none of these factors outperformed the benchmark of ‘Compulsivity and Intrusive Thought’, which was the 2^nd^ factor of a three-factor solution (Fig. 3A), as suggested by the Cattell-Nelson-Gorsuch (CNG) indices [34], a mathematical formalisation of the scree plot method [51]. We repeated this analysis for metacognitive bias, which has two opposing clinical associations – ‘Compulsivity and Intrusive Thought’ is associated with higher confidence and ‘Anxious-depression’ with lower confidence. We found that no factor outperformed the benchmark of ‘Compulsivity and Intrusive Thought’ in their positive association with metacognitive bias (i.e. higher confidence) (Fig. 3A). However, one factor, the first factor from a 2-factor solution, had a nominally stronger negative association with metacognitive bias than ‘Anxious-depression’ (Fig. 3A). The top factors had statistically significant effects on model-based planning and metacognitive bias (all *p* < 0.05) within each individual dataset (Fig. 3B). Comparing the top performing factor (the first factor from a 2-factor solution) with ‘Anxious-depression’, they were effectively identical; scores from participants in the discovery dataset (*N* = 1413) were correlated at *r*(207) = 0.99, *p* < 0.001 (Fig. 3C). Likewise, the loadings were correlated at *r*(207) = 0.94, *p* < 0.001 (Fig. 3D). Furthermore, the other factors that showed a slightly larger negative association with metacognitive bias than ‘Anxious-depression’ (specifically, the first factors from the 4-, 5-, and 6-factor solutions) were highly correlated with ‘Anxious-depression’ (all *r*_loadings_ > 0.88, all *r*_scores_ > 0.97). Additionally, scores and loadings from the second factor from the 2-factor solution was highly correlated (*r*_both_ > 0.80) with ‘Compulsivity and Intrusive Thought’ (Figure S2).

### Variation in the selection of clinical symptoms

**Fig. 4:**
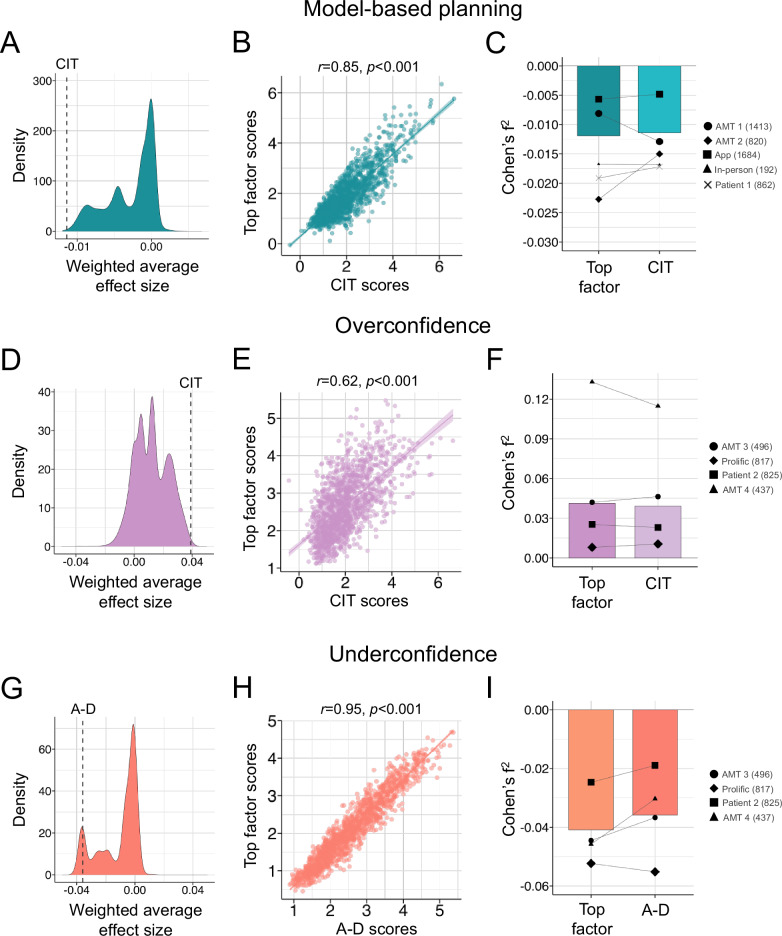
Variation in the selection of clinical symptoms. CIT Compulsivity and Intrusive Thought, A-D Anxious-depression, r Pearson correlation coefficient, p p-value. **Model-based planning. A** Density plot of effect sizes from 7665 candidate dimensions derived from all combinations of 9 questionnaires, retaining 1–5 factor solutions, predicting model-based planning. CIT was the 9^th^ best performing of 7665. **B** Final scores for the top factor and CIT correlated at r = 0.85, *p* < 0.001 within the discovery dataset (*N =* 1413). **C** Weighted average effect sizes across datasets (bars) and individual dataset effect sizes (dots) for the top factor and CIT associations with model-based planning. Overall effect sizes gains for the top factor over CIT were negligible and inconsistent across datasets. **Overconfidence. D** Density plot of effect sizes predicting overconfidence. CIT was the 23^rd^ best performing of 7665. **E** Final scores between the top factor and CIT were correlated at r = .62, *p* < 0.001 in the discovery dataset. **F** Weighted average effect sizes across datasets (bars) and individual dataset effect sizes (dots) for the top factor and CIT associations with overconfidence. Overall effect sizes gains of the top factor over CIT were once again negligible and inconsistent across datasets. **Underconfidence. G** Density plot of effect sizes predicting underconfidence. A-D was the 731^st^ best performing of 7665. **H** Final scores for the top factor and A-D were correlated at r = 0.95, *p* < 0.001 in the discovery dataset. **I** Weighted average effect sizes (bars) and individual dataset effect size (dots) for the top factor and A-D associations with underconfidence. Overall effect sizes differences between the factors were negligible, and the top factor did not consistently outperform A-D.

We conducted a multiverse analysis that systematically varied not just the number of factors retained, but the symptom feature set itself. Specifically, we conducted an independent factor analysis on all possible combinations of 1–9 questionnaire sets, considering factor solutions 1–5 for each, resulting in 7665 candidate dimensions. For their association with model-based planning, there was a large positive spike in effect sizes around 0, but the majority of factors had negative Cohen’s f^2^ effect sizes (81.03%, 6211/7665) (Fig. 4A). ‘Compulsivity and intrusive thought’ was the 9^th^ best factor for explaining individual differences in model-based planning reductions (Cohen’s f^2^ = −0.011), performing better than 99.88% of all factors generated (Fig. 4A). The top performing factor (Cohen’s f^2^ = −0.012) and ‘Compulsivity and intrusive thought’ both had highest loadings for OCD items and the lowest loadings for apathy items, with the top factor being generated from a set of questionnaires that omitted the schizotypy and alcohol addiction questionnaires (Figure S3A). Scores across the factors were highly correlated (*r*(1411) = 0.85, *p* < 0.001) in the discovery dataset (*N* = 1413) (Fig. 4B). Examining each dataset separately, the top performing factor outperformed ‘Compulsivity and Intrusive Thought’ in it’s association with model-based planning in 3/5 datasets (Fig. 4C). The top performing factor (Cohen’s f^2^ = −0.012) and ‘Compulsivity and Intrusive Thought’ (Cohen’s f^2^ = −0.011) both had very small weighted average effect sizes (Cohen’s f^2^ < 0.02) [52] (Fig. 4C). The mean difference between the weighted average effect size of the top performing factor and ‘Compulsivity and Intrusive Thought’ was not substantial in magnitude (difference in Cohen’s f^2^ = 0.001) (Fig. 4C). A heatmap of the top 1500 dimensions illustrated the relative importance of each questionnaire for explaining individual differences in model-based planning (Figure S4A). Averaging item-level loadings across the top 100 dimensions (Figure S5A), items related to OCD (M = 0.51, SD = 0.01), followed by eating disorders (M = 0.20, SD = 0.07), had the highest average loadings (Figure S5D).

‘Compulsivity and Intrusive Thought’ had a stronger association with overconfidence than 99.70% of factors (Cohen’s f^2^ = 0.039), making it the 23^rd^ top factor across analyses controlling for age, gender, IQ/levels of education and levels of ‘Anxious-depression’ (Fig. 4D). Relative to ‘Compulsivity and Intrusive Thought’, the top performing factor (Cohen’s f^2^ = 0.041) omitted the trait anxiety and eating disorder questionnaires (Figure S3B), and scores on both factors were positively correlated in the discovery dataset at *r*(1441) = 0.62, *p* < 0.001 (Fig. 4E). While the effect size was nominally greater for the top performing factor across samples, this difference was miniscule (Cohen’s f^2^ difference = 0.002), and the top factor did not outperform ‘Compulsivity and Intrusive Thought’ within half (2/4) of the datasets (Fig. 4F). Items related to OCD, schizotypy and impulsivity had strong positive loadings across the top 1500 performing factors (Figure S4B) and specifically, items from the OCD questionnaire contributed most predominantly to the top 100 dimensions associated with overconfidence (Figure S5B), and had the highest questionnaire-level average loadings (M = 0.36, SD = 0.02) (Figure S5D).

When examining negative associations with confidence bias, ‘Anxious-depression’ was the 731^st^ best performing factor (better than 90.46% of factors) in terms of its association with confidence (Cohen’s f^2^ = −0.036) controlling for age, gender, IQ/levels of education and levels of ‘Compulsivity and Intrusive Thought’ (Fig. 4G). ‘Anxious-depression’ scores were highly correlated at (*r*(1411) = 0.95, *p* < 0.001), with the top performing factor (Cohen’s f^2^ = −0.041) (Fig. 4H), which had high loadings for the trait anxiety items (Figure S3C). Despite having a larger negative effect size across samples, the top generated factor did not consistently outperform ‘Anxious-depression’, which had a larger effect size in the Prolific dataset (*N* = 817) (Fig. 4I). Overall, the differences in effect size between the top performing dimensions versus ‘Anxious-depression’ were negligible, reflecting a weighted effect size difference of r = 0.005 for underconfidence (Fig. 4I). Visualising the heatmap of item loadings across the top 1500 factors, trait anxiety contributed highly to explaining individual differences in underconfidence (Figure S4C), and had the highest average item- and questionnaire-level loadings across the top 100 dimensions (M = 0.59, SD = 0.02) (Figure S5C-D).

### Higher- versus first-order factor rotation

Next we examined how factor rotation relate to the fit of dimensions to cognition, comparing orthogonal, oblique rotations and a higher order solution, which includes a general hierarchical (‘p’) factor. The cross-solution correlations (Figure S7A) of scores were very high across oblique (oblimin) and orthogonal (varimax) first-order solutions, but the oblique rotation (2nd factor – ‘Compulsivity and Intrusive Thought’) produced the nominally largest association with reductions in model-based planning (Cohen’s f2 = −0.014) across our 5 datasets (Figure S7B). The same was true for both under- and over-confidence in metacognitive biases. ‘Compulsivity and Intrusive Thought’ showed the largest positive association (Cohen’s f2 = 0.038) and ‘Anxious-depression’ the largest negative association (Cohen’s f2 = −0.030) (Figure S7B). Notably, the general (‘p’) factor from the bifactor model showed the relatively smaller associations with all three cognitive domains (Figure S7B). Zooming in on this, associations with the ‘p’ factor were not statistically significant within any of the datasets for model-based planning (all *p* > 0.05). The ‘p’ factor was associated with metacognitive bias in two datasets, but these were in opposing directions (negative association in the Prolific, *N* = 817 and positive association in the AMT 4, *N* = 437 datasets) (Figure S7C).

### Comparison to a supervised method to derive factors

**Fig. 5:**
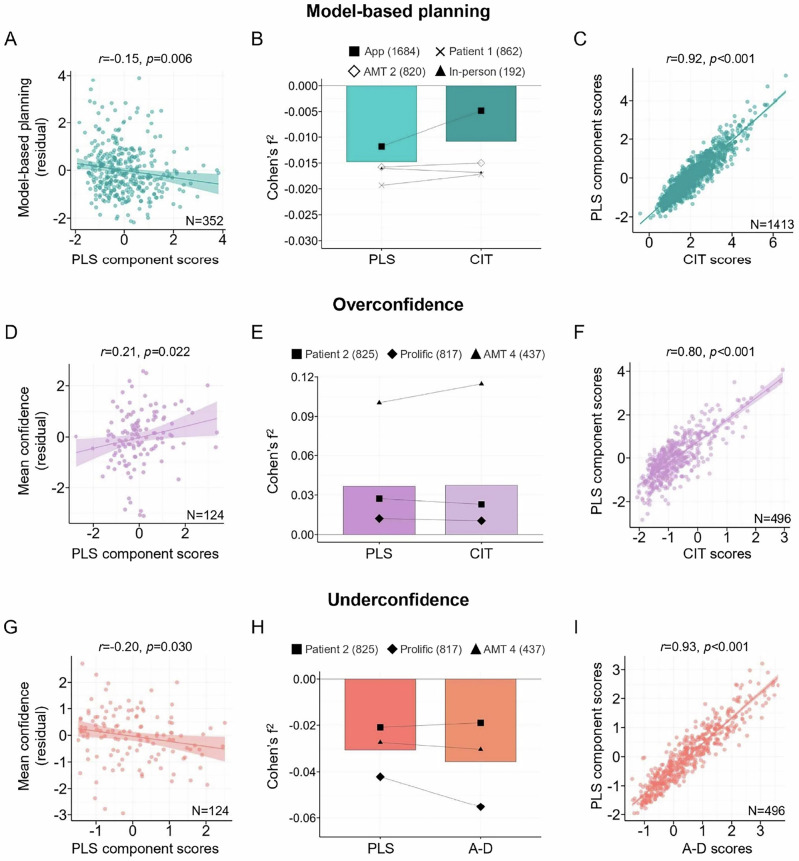
Partial Least Squares Regression Analyses of Model-based Planning and Metacognitive Bias. PLS Partial least squares regression, CIT Compulsivity and Intrusive Thought, A-D Anxious-depression. **Model-based planning.** **A** The correlation between PLS component scores and the residual of model-based planning (controlling for age, gender and IQ) in the held out 25% of the data (*N =* 352). **B** Weighted average effect sizes across out-of-sample datasets (bars) and individual dataset effect sizes (dots) for the PLS component and CIT associations with model-based planning. The overall gain in the weighted average effect size for the PLS component was negligible and inconsistent across datasets. **C** Correlation between PLS component scores and CIT scores in the full discovery dataset (*N =* 1413). **Overconfidence.** **D** The correlation between PLS component scores and the residual of confidence (controlling for age, gender, IQ and A-D) in the held out 25% of the data (*N =* 124). **E** Weighted average effect sizes across out-of-sample datasets (bars) and individual dataset effect sizes (dots) for the PLS component and CIT associations with confidence. The weighted average effect size of the PLS component was not nominally greater than the CIT effect, and the PLS component only outperformed CIT  (nominally) in 2/3 datasets. **F** Correlation between PLS component scores and CIT scores in the full discovery dataset (*N =* 496). **Underconfidence.** **G** The correlation between PLS component scores and the residual of confidence (controlling for age, gender, IQ and CIT) in the held out 25% of the data (*N =* 124). **H** Weighted average effect sizes across out-of-sample datasets (bars) and individual dataset effect sizes (dots) for the PLS component and AD associations with confidence. The weighted average effect size was higher for A-D compared to the PLS component and A-D performed better in 2/3 datasets. **I** Correlation between PLS component scores and AD scores in the full discovery dataset (*N =* 496).

The preceding analyses focused on the extent to which natural patterns of symptom covariation possess mechanistic validity. In a final analysis, we compared the resulting factors to those derived using a fully supervised approach, where cognitive performance (i.e., model-based planning and metacognitive bias) informs the selection of the factor itself. Specifically, we used partial least squares regression with 10-fold cross-validation within 75% of the discovery dataset [34] to first identify a transdiagnostic component linked to individual differences in model-based planning (Figure S8A and Table S1). Across the test folds of the cross-validation procedure run on the discovery set (*N* = 1061), higher component scores were associated with model-based planning (residualised for age, gender and IQ) with an average r^2^ = 0.05 in the test folds of the cross-validation. Evaluating the model in the held out 25% of the data from the discovery sample (*N* = 352), the PLS component score was significantly negatively associated with the residual of model-based planning abilities, *r*(350) = −0.15, *p* = 0.006 (Fig. 5A). Testing model performance out-of-sample, the PLS component performed was higher than ‘Compulsivity and Intrusive Thought’ in 3 of the 4 independent datasets (Fig. 5B). However, the weighted average effect size for the PLS component (Cohen’s f^2^ = −0.015) was only slightly larger than the effect size for ‘Compulsivity and Intrusive Thought’ (Cohen’s f^2^ = −0.011), with an average gain in effect size of f^2^ = 0.004 (Fig. 5B). In the full discovery dataset (*N* = 1413), scores on the PLS factor were strongly correlated with ‘Compulsivity and Intrusive Thought’, *r*(1411) = 0.92, *p* < 0.001 (Fig. 5C).

A PLS component was generated to predict individual differences in overconfidence within the AMT 3 sample (*N* = 496) [40] (Figure S8B and Table S1). The PLS component was associated with the residual values of confidence (after controlling for age, gender, IQ and levels of ‘Anxious-depression’) at *r*^2^ = 0.18, averaged across the cross-validation test folds in the training 75% of the data (*N* = 372). The PLS component was significant positively associated with mean confidence in the held out 25% of the data (*N* = 124), *r*(122) = 0.21, *p* = 0.022 (Fig. 5D). With out-of-sample testing, the weighted average effect size of the PLS component (Cohen’s f^2^ = 0.037) was nominally smallerer than the effect of ‘Compulsivity and Intrusive Thought’ (Cohen’s f^2^ = 0.037) when predicting individual differences in overconfidence across all 3 independent datasets (Fig. 5E). Within the full discovery dataset (*N* = 496), scores on the PLS component were strongly correlated (*r*(494) = 0.80, *p* < 0.001) with ‘Compulsivity and Intrusive Thought’ scores (Fig. 5F). We repeated this analysis residualising ‘Compulsivity and Intrusive Thought’, instead of ‘Anxious-depression’, from the confidence measure to identify a PLS component significantly negatively associated with confidence (Figure S8C and Table S1). This component had an average r^2^ = 0.07 in the training set (*N* = 372) and was negatively associated with the residual of confidence in the held out 25% of the data (*N* = 124), *r*(122) = −0.20, *p* = 0.030 (Fig. 5G). The PLS component did not outperform ‘Anxious-depression’ in 2 of the 3 out-of-sample tests, and had a lower weighted average effect size (Cohen’s f^2^ = −0.031) compared to ‘Anxious-depression’ (Cohen’s f^2^ = 0.036) (Fig. 5H). Scores for the PLS component factor were highly correlated (*r*(494) = 0.93, *p* < 0.001) with ‘Anxious-depression’ scores (Fig. 5I).

## Discussion

There is broad agreement that current diagnostic categories present challenges for mechanistic research in psychiatry [53, 54]. There is less agreement, however, about how alternative classification frameworks should be generated. Some have suggested that correlation at the level of symptoms can reveal an important latent structure of mental health, which is statistically robust and reproducible [25, 26, 47]. Others have argued that this approach may be fraught, because there is no reason to think that a descriptive construct, albeit one that is statistically valid, has any specific underlying biological mechanism [27, 55]. This issue is compounded by the analytic degrees of freedom inherent in such correlation-based approaches, wherein researcher choices at the time of data collection and analysis can produce many alternative factor solutions.

Here we conducted an exhaustive multiverse analysis of potential factor solutions and tested their mechanistic validity, operationalised as their association with three aspects of cognition. In nine datasets, comprising *N* = 7565 individuals, we provide evidence that those factors with the greatest mechanistic validity converge on the factors derived from a first-order factor analysis with oblique rotation, applied to a transdiagnostic sets of the maximal number of theoretically informed self-report symptoms available to the researcher. These canonical factors (‘Compulsivity and Intrusive Thought’ and ‘Anxious-depression’) performed better than 99 and 90% (respectively) of alternatives in their association with cognition. These factors consistently out-performed total scores on constituent questionnaires and, in some cases, dimensional effects were twice as large as classic clinical questionnaire effects. In cases where they performed nominally worse than the top-performing factor from multiverse analyses, the differences in effect size were trivial, inconsistent across datasets and correlations between competing factors approached unity. Perhaps most surprisingly, we showed that dimensions derived from factor analysis at the symptom-level had as strong an association to cognition as dimensions derived from a supervised approach that expressly maximised the association between a factor and cognitive performance. This suggests that novel dimensions of psychopathology can be identified using symptoms alone, opening the door to leveraging other large existing datasets, without cognitive test data, to identify novel transdiagnostic dimensions. Our results generalised across nine independent samples with markedly different characteristics, including patients about to start treatment, the general population, paid and unpaid participants, of different ages, collected in-person, online or in-app, and using several variants of the cognitive tasks in question. This remarkable stability across heterogeneous datasets suggests that factor solutions generated based on the correlation of experienced symptoms are a valid and powerful approach for defining novel transdiagnostic dimensions for research in psychiatry.

As part of this multiverse analysis, we showed that a first-order correlated factors model outperformed a bifactor model. The ‘p’ factor has garnered significant interest in psychiatric research since its initial identification, and has been suggested by some to reflect the liability, comorbidity, persistence, and severity of psychopathology [31]. It features prominently in the HiToP framework as a ‘superspectrum’, and although it is without doubt a statistical feature of mental health questionnaire items, concerns have been raised about the interpretation of the ‘p’ factor [56, 57], and more broadly the evaluation and interpretation of bifactor models, including model overfitting [58, 59], and poor replication across samples [60]. Across the nine datasets and their aggregates, we found no association between the ‘p’ factor and any of the three cognitive phenotypes under study. These results suggest two things. First, it suggests that the ‘p’ factor, if it constitutes an important level of clinical description, may reflect nonspecific psychopathology [31]. Second, the ‘p’ factor is near indistinguishable from the sum of the feature matrix that generates it [57], and so the finding that it is unrelated to cognitive test performance further underscores that the specificity of associations we report between cognition and ‘Anxious-depression’ and ‘Compulsivity and Intrusive Thought’.

Computational Factor modelling and the HiTOP approach share many features, agreeing most notability in the value of a transdiagnostic dimensional approach and using the covariance of symptoms to derive it. There are some points of distinction however. Computational Factor modelling places a premium on external validation with the goal of explaining mechanisms of illness (e.g. with cognitive computational tasks), while HiTOP focuses more simply on the structure of mental health. Based on the results of the present study, that self-report covariation provides a similar result to the supervised mechanistic approach, it appears likely that the two approaches may dovetail in the near future. One reason that they do not yet align, in terms of the specific dimensions identified, is that the frameworks have focused on different aspects of mental health (with Computational Factor modelling being more narrow). Perhaps more importantly, HiTOP factor analyses have been historically based on DSM-diagnoses, and Computational Factor modelling uses raw self-report data of individual symptoms [25]. HiTOP have begun to move in this direction, and a recent bottom up and data driven analysis of self-report versions of DSM symptoms (rather than disorders) found that repetitive rituals and compulsions loaded onto a positive psychosis factor, consistent with Computational Factor modelling but not the original HiTOP formulation [61]. This provides further evidence that the two systems may converge in time. Conceptually, Computational Factor modelling also aligns with the RDoC initiative, which also emphasises biobehavioural and neurocognitive processes over traditional diagnostic labels; however, RDoC is explicitly a research framework and does not fully describe psychiatric phenotypes [24]. Integrating RDoC constructs can strengthen Computational Factor modelling by informing the selection and interpretation of mechanistic dimensions. Together, this combined strategy, starting with symptom covariance, refining dimensions through cognitive association, and grounding them in neurocognitive constructs like those in RDoC, offers a promising path toward a more mechanistically valid nosology for psychopathology.

Mechanistic validity is just one way in which we might evaluate a candidate classification system in psychiatry. Alternative objective functions, for example maximising discriminative treatment-prediction, could produce different dimensions that are of more immediate clinical value [55], even if they provide little-to-no insights with respect to aetiology or brain-behaviour correspondence. We do not see these as competing goals, per se, but rather different levels of analysis. The current focus on improved mapping of symptom dimensions to neurocomputational mechanisms is designed to provide a new, theory-based path to understanding mental health and illness, that could lead to the development of precision therapeutics that target cognitive processes or early detection [28]. Future research should test if treatment-oriented vs. mechanistic approaches to symptom validation are distinct and complementary or converge on the same or similar factors. The present study cannot speak directly to this potential. However, there is some preliminary evidence that transdiagnostic dimensions derived from Computational Factor modelling may help us understand how treatments may work. For example, metacognitive biases diminish following treatment with cognitive behaviour therapy and those with the largest increases in metacognitive confidence following treatment had the greatest reductions in ‘Anxious-depression’ [43]. To further elucidate the causal mechanism of this effect, more intensive repeated testing would be a promising approach to account for temporal and contextual variation [62]. Unlike metacognitive bias, reductions in model-based planning are more trait-like; they do not improve following successful treatment and may instead confer vulnerability to disorders of compulsion [63, 64].

While the top performing factors had considerably stronger cognitive correlates than individual questionnaire total scores, the average effect sizes remained small in magnitude overall. This underscores how individual cognitive tests can at best provide a highly simplistic mechanistic account of psychopathology [21]. For a comprehensive explanatory model, multivariable models are required, incorporating micro (e.g., neural circuits) and macro (e.g., societal factors) levels of information [65]. An additional limitation of the current study is that we only considered nine specific clinical questionnaires for practical reasons (i.e. data availability), limiting coverage of the broader symptom spectrum. Future work aiming to capture the full spectrum of psychopathology must consider additional facets of mental health (e.g., symptoms of autism spectrum disorder or attention deficit hyperactive disorder [66]) and aspects of cognition specifically relevant to them. An important consideration when interpreting the results of factor analytic models applied to multiple questionnaires is that each questionnaire has often been optimised for internal consistency and diagnostic specificity. As a result, the latent structures may in part reflect the design constraints of the questionnaires themselves. This was evident in our results, where at a certain point, factor analysis tended to recover constructs closely aligned with individual questionnaires. One potential solution to this may be redesigning questionnaires to remove superficial features that might be sources of variance not meaningfully related to mental health. In addition, incorporating independent data modalities for external validation of phenotypes will be important. This might include other cognitive measures, but also neuroimaging, life history and ambulatory assessments. It is possible that different levels of a dimensional hierarchy may map onto different sources of data, e.g., some symptoms that co-occur might do so due to common environmental factors, but have distinct neural or behavioural correlates [67, 68]. As cognitive test performance is but one layer of mechanistic precision – future work aiming to link brain-based measures to these dimensions may reveal stronger associations than the existing literature has been able to [36, 69], providing a more compelling path towards novel pharmacological or stimulation-based interventions. That said, the strong convergence observed across three aspects of cognition studies in the present study provides a promising foundation upon which to further examine the generality of the conclusions drawn.

In conclusion, we used data-driven multiverse analysis of large-scale datasets to identify dimensions of psychopathology with optimal mechanistic validity. The natural covariance patterns of symptoms corresponded to specific aspects of cognition, and these were largely robust to manipulations of the number of factors retained, various combinations of questionnaire sets and rotation type. Specifically, dimensions that maximally corresponded to distinct cognitive capacities were generated through first-order factor analysis, with oblique rotation, a factor number indexed by the scree plot and a broad set of theoretically-informed clinical questionnaires. This demonstrated that Computational Factor modelling can derive dimensions of psychopathology that are clinically sensible and cognitively meaningful.

## Methods

### Participants

In total, nine datasets were included in this study (Fig. 1A). Five of these datasets included measures of model-based planning [33–36, 50], and four included measures of metacognitive bias [39, 40, 42, 43]. Each study gathered informed consent from participants, including for data re-use, and obtained ethical approval from a local institutional review board. *N* = 1413 individuals were included in the discovery sample, previously described in Gillan et al. [34] (labelled AMT 1, to distinguish it from other AMT samples). We chose this dataset as the discovery sample, as this study was the first to derive the established transdiagnostic dimensions and is the sample from which the other studies have applied the factor weights to generate independent dimension scores, making it a widely accepted reference point in the literature. Additionally, in early exploratory analyses, we found that the factor structures obtained from other datasets (including the largest dataset available and the clinical sample) were highly similar to those from the Gillan et al. [34] dataset, suggesting that varying this step would not substantially alter the results (see ‘Extracting factor weights from discovery dataset’ below). For consistency and ease of interpretation, and given the strong precedent set by prior work, we used this dataset to generate the factors. Individuals in this samples were recruited remotely, online through AMT [34]. The sample had a mean age of 32.97 (SD = 10.81), was mostly female (*n =* 823, 58.20%), with a mean IQ of 98.00 (SD = 9.55) (Table 1).

**Table 1:** Sociodemographic Characteristics.

	Model-based planning					Metacognitive bias			
Socio-demographic characteristic	AMT 1 (*N* = 1413)	Smartphone App (*N* = 1684)^a^	AMT 2 (*N* = 820)	Student (*N* = 192)	Patient 1 (*N* = 862)	AMT 3 (*N* = 496)	AMT 4 (*N* = 437)	Patient 2 (*N* = 825)	Prolific (*N* = 817)
Gender, *N (%)*									
Male	590 (41.80)	483 (28.68)	421 (51.34)	80 (41.67)	178 (20.65)	256 (51.61)	241 (55.15)	173 (20.97)	331 (40.51)
Female	823 (58.20)	1163 (69.06)	399 (48.66)	112 (58.33)	676 (78.42)	240 (48.39)	196 (44.85)	644 (78.06)	486 (59.49)
Other		36 (2.14)			8 (0.93)			8 (0.97)	
Age, *M* (*SD*)	32.97 (10.81)	46.14 (14.70)	34.89 (10.05)	31.89 (12.10)	32.07 (11.06)	35.59 (10.57)	37.54 (10.39)	32.00 (11.02)	25.58 (9.79)
IQ, *M* (*SD*)	98.00 (9.55)		99.00 (9.74)	7.95 (3.29)		7.98 (3.47)	−0.27 (0.79)		
Education, *N (%)*									
Below undergraduate		609 (36.16)			203 (23.55)			196 (23.76)	
Completed undergraduate		678 (40.26)			453 (52.55)			433 (52.48)	
Above undergraduate		395 (23.46)			206 (23.90)			196 (23.76)	

*AMT* amazon’s mechanical turk, *IQ* intelligence quotient, *M* mean, *SD* standard deviation, *N* count.

^a^missing data for 2 participants.

The other samples with measures of model-based planning included three previously published datasets [33, 35, 36] and unpublished data from the Precision in Psychiatry (PIP) study [50]. Participants in the Patzelt et al. [35] dataset were recruited online using (*N* = 820) (labelled AMT 2). We had a slightly lower sample than the sample published in the Patzelt et al. [35] paper, as we only included those with full model-based planning, questionnaire and sociodemographic information. Participants in the Seow et al. [36] dataset were from the general population and recruited in-person (*N* = 192). The Donegan et al. [33] dataset included participants from the Neureka Project, which enrols members from the general public who voluntarily download a smartphone application to contribute to scientific research. We had a slightly larger sample (*N* = 1785) than the sample published in Donegan et al. [33] paper, as we included additional Neureka users who have completed the model-based planning task since the study publication. The PIP study recruited participants from clinical sites that made referrals for internet-based cognitive behavioural therapy [50]. Among PIP study completers, *N* = 862 completed a model-based planning task [50] (labelled Patient 1), and *N* = 825 completed the metacognitive task [43] (labelled Patient 2). The sample size in this paper (*N* = 825) is larger than Fox, Lee, et al. [50], as we included participants excluded from that study for not completing a follow-up assessment. Three of the other datasets with measures of metacognitive bias were pre-existing, published datasets, that recruited participants online through crowdsourcing AMT (Rouault et al., [40], *N* = 496 (labelled AMT 3) and Seow & Gillan, [42], *N* = 437 (labelled AMT 4)), or Prolific (Benwell et al., [39], *N* = 817). The sociodemographic descriptives for all datasets are included in Table 1.

#### Ethics approval and consent to participate

The study included nine independent samples, each obtained under separate ethical approvals [33–36, 39, 40, 42, 43, 50]. Approvals were granted by the Research Ethics Committee of the School of Psychology at Trinity College Dublin [33, 36, 42, 43, 50] (Project IDs: SPREC072019-01 [33]; SPREC072017-01 [43, 50]), the New York University Committee on Activities Involving Human Subjects [34], the Harvard Committee on the Use of Human Subjects [35], the University of Dundee Research Ethics Committee [39], the University College London Research Ethics Committee (Project ID 1260/003) [40], and the Northwest–Greater Manchester West Research Ethics Committee of the NHS, Health Research Authority, and Health and Care Research Wales (REC 20/NW/0020, Project ID 270623) [43, 50]. All methods were performed in accordance with relevant guidelines and regulations. Informed consent, including for data re-use, was obtained from all participants. Full ethical details for each dataset are reported in the corresponding published papers [33–36, 39, 40, 42, 43, 50].

### Procedures

#### Self-report clinical questionnaires

In each study, participants completed 209 items from nine self-report clinical questionnaires that assess a variety of psychiatric symptoms, including depression (Zung Self-Rating Depression Scale) [70], trait anxiety (State Trait Anxiety Inventory) [71], schizotypy (Short Scales for Measuring Schizotypy) [72], impulsivity (Barratt Impulsiveness Scale 11) [73], OCD (Obsessive-Compulsive Inventory-Revised, OCI-R) [74], social anxiety (Liebowitz Social Anxiety Scale) [75], eating disorders (Eating Attitudes Test) [76], apathy (Apathy Evaluation Scale) [77], and alcohol misuse (Alcohol Use Disorders Identification Test) [78]. These questionnaires were chosen based on prior factor analysis in the discovery sample study [34], which demonstrated that these items could be used to generate the established ‘Anxious-depression’ and ‘Compulsivity and Intrusive Thought’ dimensions previously associated with model-based planning and metacognitive bias. The core dataset of 209 items is by no means exhaustive of mental health; it comes from the original work developing the method of computational factor analysis [34]. The items were drawn from well-validated self-report scales covering a broad range of symptom domains that were predicted to have convergent and divergent associations with one aspects of cognition - model-based planning – in the original study. We focused on them here due to the unique opportunity of there existing several large, diverse and independent samples using the same item set and variants of the same tasks which allow us to perform data-driven modelling and robust out of sample validation.

#### Model-based planning tasks

Alternative versions of the reinforcement-learning ‘two-step’ task [32, 79] were used in the five samples that measured model-based planning. In the two-step task used to quantify model-based planning, participants are presented with a series of choices between two stimuli (often represented as abstract symbols or images). Each choice leads to another set of options, creating a two-step decision-making process (Fig. 1A). The key feature of the two-step task is that the outcomes of the initial choices are probabilistically associated with different outcomes in the subsequent steps. Participants must learn these associations through trial and error. Model-based learning refers to the ability to learn and utilize an explicit model of the task structure to make decisions. In the context of the two-step task, this involves understanding the probabilistic relationships between choices and outcomes and using this knowledge to plan and select actions that are expected to yield favourable outcomes in the long run. More detailed descriptions of the adapted two-step task versions are included in the original publications for each study [33–36, 50].

#### Metacognitive tasks

Metacognitive bias was measured through adapted versions of a visuo-perceptual decision-making task [39, 40, 43], a knowledge decision-making task [39], or a predictive inference task [42]. In the visuo-perceptual decision-making tasks, participants make a choice as to which of two stimuli contains more dots, and then subsequently rate their confidence in the accuracy of their choice, across multiple trials (Fig. 1A). The knowledge-based decision-making task requires participants to repeatedly judge which of two countries has the larger human population and then provide a confidence rating for their judgment. In contrast, the predictive inference task involves participants aiming a particle from the centre of a large circle to hit a target. Participants then rate their confidence that the particle would hit the target. Detailed descriptions of the tasks can be found in the prior publications from which the datasets were taken [39, 40, 42, 43].

### Data preparation and analysis

#### Quantifying model-based planning

The ‘two-step’ task can be used to assess an individual’s model-based planning by using logistic regression analyses with mixed-effect models to predict their choice on the subsequent trial. Specifically, model-based planning was indexed as the interaction effect of reward and transition on their choice stochasticity [33, 34, 36, 50]. Alternatively, model-based planning was calculated as the fit of a computational learning model, in which choices were reflected as the weighted combination of model-free and model-based planning [35]. For all datasets, reductions in model-based planning were indicated by lower model-based learning values.

#### Quantifying metacognitive bias

Explicit post-decisional confidence judgements were used to measure metacognition across the four datasets [39, 40, 42, 43]. Metacognitive bias was calculated as the mean confidence reported by participants across experimental trials. Higher mean confidence, relative to within-sample estimates, was used to index ‘overconfidence’, while ‘underconfidence’ was indicated as relatively lower mean confidence.

#### Extracting factor weights from discovery dataset

Using the self-reported clinical questionnaire data from the Gillan et al. [34] dataset only, we created a multiverse of candidate factor solutions by iterating over the number of factors retained, the sets of questionnaire items analysed, and the rotations implemented (Fig. 1B). We used a single discovery dataset to derive candidate factor solutions and applied the weights to the other datasets. This allowed direct comparison of identical factors across datasets. The factor solutions derived from the other large datasets were however highly similar. For example, the correlation of weights between the discovery dataset and an independent factor analysis conducted on the largest dataset (Neureka) ranged from r = 0.94 to r = 0.95 for the 3-factor solution and from the patient sample the similarities ranged from r = 0.84 to r = 0.96.

#### Variation of the number of factors selected for retention

First, we generated a heterogenous correlation matrix of the 209 items from the nine questionnaires using the hector function in the polycor package in R. We conducted maximum likelihood estimation (MLE) factor analysis using the fa function from the psych package in R. We specified that each iteration would run from a single to 10-factor structure, using regression with an oblique rotation. The oblique rotational ‘oblimin’ was used, consistent with prior factor analysis of this questionnaire set [34]. Factor analysing each factor number from a single to 10-factor structure generated factor weights and loadings for 55 candidate dimensions in total (Fig. 1B(i)).

#### Variation in the selection of clinical symptoms

Candidate dimensions for multiverse analysis were identified in the Gillan et al. [34] sample by factor analysing every possible combination of the nine clinical questionnaires. Combinations ranged from each questionnaire on its own, to all nine questionnaires considered together, giving a total of 511 possible combinations (Fig. 1B(ii)). For each of the standalone combinations of questionnaires, we generated a heterogenous correlation matrix for the items included in that combination. The correlation matrices were then factor analysed (MLE in the fa package in R), with the oblique rotational ‘oblimin’. Rather than using the scree plot to determine factor number, we extracted 1-factor, 2-factor, ... up to 5-factor solutions for each of the 511 sets of items. Factor analysing each combination with up to 5 potential factors generated factor weights and loadings for 7665 candidate dimensions in total Fig. 1B(ii).

#### Higher- versus first-order factor rotation

The heterogenous correlation matrix of the full 209 item set was factor analysed (MLE in the fa package in R), specifying a three-factor structure. A three-factor structure was chosen following its strong performance in the analysis comparing 1-10 factor solutions (‘variation in the number of factors selected for rotation’). We considered 3 commonly employed rotation types: the oblique rotation, ‘oblimin’ (used in the prior sections), and orthogonal rotations, ‘varimax’, and a ‘bifactor’ solution (Fig. 1B(iii)). While the oblimin and varimax rotations were generated using the fa package in R, the bifactor solution was calculated using the ‘omega’ function from the Psych package in R, as per prior research on bifactor modelling in psychiatry [80]. The within-solution correlations between factor scores verified the oblique and orthogonal nature across rotation types (Figure S6). Factor analysing each rotation type with a three-factor structure (with an additional hierarchical general ‘p’ factor for the bifactor solution) produced weights and loadings for 10 candidate dimensions in total (Fig. 1B(iii)).

#### Testing candidate dimensions across datasets

Considering each manipulation type separately (factor number, combinations of scales and rotation type), we calculated the factor scores for each participant within each of the eight datasets separately. Participants’ factor scores were calculated as the sum of the item weight by participants’ response to that item, for each factor (i.e., dimension score = sum(item response*weight)) (Fig. 1C(i)). This only differed slightly for our bifactor model, as the ‘omega’ output does not provide factor weights. To account for this, factor scores for our bifactor model were calculated from the factor loadings using the Anderson-Rubin method, in which a least-squares formula is applied to maintain the orthogonality of the general and specific factor scores [81].

We then ran linear regression analysis to determine the association between neurocognitive abilities (model-based planning/metacognitive bias) and factors scores, controlling for age, gender and IQ/education. Age, gender and IQ/education were included as covariates to account for their potential effects on model-based planning and confidence. For each regression analysis, we calculated the effect size of each factor as Cohen’s f^2^ value [52] (Fig. 1C(ii)). For the factor number and rotation type manipulations, our regression models included all the dimensions generated within that factor structure. For example, the regression model with a three-factor structure would be: model-based planning/metacognition ~ factor 1 scores + factor 2 scores + factor 3 scores + age + gender + IQ/education (Fig. 1C(ii)). We included all factors within the structure in the same model, as the dimensions across the factor structure are obliquely rotated, meaning that factor scores are related across the structure. Including all factors means the model accounts for the effects of the related factors. This is more important for metacognition, as it has bidirectional clinical associations (positively linked to ‘Compulsivity and Intrusive Thought’ but negatively linked to ‘Anxious-depression’). For the interested reader, see supplementary Figure S2 for sensitivity analyses showing individual model results without these controls, where overall effect sizes are smaller. When we tested the impact of the inclusion/exclusion of questionnaires (i.e. ‘Variation in the selection of clinical symptoms’), we only include individual factor scores in each of the 7665 models predicting model-based planning. For metacognitive bias, we included ‘Anxious-depression’ as an additional covariate to determine which factors explained the most variance in overconfidence. When we were interested in factors that predicted underconfidence, conversely, we reran the models and included ‘Compulsivity and Intrusive Thought’ instead of ‘Anxious-depression’ as a covariate.

Following this, we took each dataset with a measure of model-based planning and calculated the weighted (by sample size) average Cohen’s f^2^ for each factor across the samples, consistent with a meta-analytic approach. We then repeated this separately for each dataset that had a measure of metacognitive bias to get the weighted average Cohen’s f^2^ when mean confidence was the dependent variable. The weighted average Cohen’s f^2^ were used to determine which factor performed best when predicting individual differences in model-based planning and metacognitive bias (i.e., which factors were the winning solutions). For model-based planning, we were specifically interested in reductions (lowest Cohen’s f^2^ values). For metacognitive bias, we were interested in both directions (highest and lowest Cohen’s f^2^ values, measuring over- and under-confidence respectively) (Fig. 1C(iii)).

#### PLS regression

We used partial least squared regression to identify transdiagnostic latent dimensions, comprised of the 209 questionnaire items, which are linked to individual differences in cognitive outcomes. The AMT 1 sample (*N* = 1413) [34] was used as discovery datasets to generate the PLS component for model-based planning, as this sample was also used to discover the factor structures from unsupervised factor analysis. For metacognitive bias, the AMT 3 sample (*N* = 496) [40] were used as discovery datasets to generate the PLS component. The Rouault et al. [40] sample was chosen because it is the largest dataset available with a general population sample and data on metacognition and all covariates (age, gender and IQ). To avoid model overfitting, we split each discovery dataset into training and test sets, comprising of 75 and 25% of the data, respectively [82]. To identify the optimal number of components and equivalent item loadings for components within the training set, we used a 10-fold cross-validation procedure, fitting the model on 90% and testing performance on the left-out 10% of the data. The mean squared error of the model’s predictions was then averaged across test folds to provide an index of the model’s predictive accuracy with different numbers of components. Within the training set, the optimal models for model-based planning (RMSE = 0.99), overconfidence (RMSE = 0.98) and underconfidence (RMSE = 0.98) all contained a single component. The item loadings for components were used to generate participants’ dimension scores (dimension score = sum(item response x loading)). We then evaluated if component scores were significantly associated with cognitive outcomes within the training and test sets separately, before testing model performance out of sample.

## Supplementary information

Supplementary Material

## Data Availability

Researchers may access the data by completing and submitting a Data Request Form for Research Purposes at https://osf.io/5ajp4/.

## Code availability

The R analysis scripts are available at https://osf.io/5ajp4/.
