# Supplementary material for: Cognitive arbitration between candidate dimensions of psychopathology
Source: Mol Psychiatry. 2025 Oct 15;31(3):1634–47. doi: 10.1038/s41380-025-03297-2 (PMC12916499; doi:10.1038/s41380-025-03297-2)
Supplement: Supplementary file 1 — Supplementary Material [file 41380_2025_3297_MOESM1_ESM.pdf]

Cognitive arbitration between candidate dimensions of psychopathology

Supplementary Information

Celine A Fox[1,2], Vanessa Teckentrup[1,2], Kelly R Donegan[1,2], Tricia XF Seow[3,4], Christopher SY Benwell[5], Brenden Tervo-Clemmens[6], Claire M Gillan[1,2]*

[1]School of Psychology, Trinity College Dublin, Dublin, Ireland
[2]Trinity College Institute of Neuroscience, Trinity College Dublin, Dublin, Ireland
[3]Functional Imaging Laboratory, University College London, London, UK
[4]Max Planck UCL Centre for Computational Psychiatry and Ageing Research, University College London, London, UK
[5]Division of Psychology, School of Humanities, Social Sciences and Law, University of Dundee, Dundee, UK
[6]Department of Psychiatry & Behavioral Sciences, University of Minnesota, Minnesota, USA

* = Claire Gillan is the corresponding author

Table of Contents

RESULTS **3**
Variation in the number of factors retained 3
Figure S1. Re-analyses of variation in the number of factors retained, without controlling for other factors from that model.. 4
Figure S2. Re-analyses of variation in the number of factors retained, without controlling for other factors from that model. 5
Variation in the selection of clinical symptoms 6
Figure S3. Top (1/7665) performing dimensions from varying the selection of clinical symptoms 7
Figure S4. Heatmaps of top 1500/7665 performing dimensions from varying the selection of clinical symptoms 8
Figure S5. Average across top 100/7665 performing dimensions from varying the selection of clinical symptoms.. 9
Higher- versus first-order factor rotation 10
Figure S6. Higher- versus first-order factor rotation: The within-solution correlations between factor scores 10
Figure S7. Higher- versus first-order factor rotation 10
Partial Least Squares Regression 12
Figure S8. Weights of items across components generated with Partial Least Squares Regression 12
Table S1. Individual questionnaire items and weights for the Partial Least Squares Regression solutions, Anxious-depression and Compulsivity and Intrusive Thought 13

RESULTS

Variation in the number of factors retained

To compare the 55 factors generated from a single to 10-factor solution (Figure 3A), we reran regression models per task, including each factor within independent models, along with age, gender and education/IQ. Replicating the findings when factor scores from each solution were included in the same model (Figure 3A), no factor outperformed the benchmark of ‘Compulsivity and Intrusive Thought’ for the association with model-based planning (Figure S1). For associations with metacognitive bias, we found that no factor outperformed the benchmark of ‘Compulsivity and Intrusive Thought’ in terms of a positive association with metacognitive bias (i.e., higher confidence) (Figure S1), again replicating the top performance of ‘Compulsivity and Intrusive Thought’ when factors from the same solution were included in the same model (Figure 3A). The first factor from a 4-factor solution had a stronger negative association with metacognitive bias than ‘Anxious-Depression’ (Figure S1), which contrasted the top effect of the first factor in a 2-factor model when all solution factors were included in the regression model (Figure 3A). Notably, including the factors from each solution in separate regression models produces top effects that are relatively smaller in magnitude than the effects from models that include all factors within a solution, especially for metacognitive bias (magnitude of effects in Figure 3A vs Figure S1). For example, the magnitude of the effect of ‘Compulsivity and Intrusive Thought’ on confidence bias is nominally larger when factors from the same solution are included in the model (Cohen’s f^2= 0.037, Figure 3B) compared to when the other factors are not controlled for (Cohen’s f^2=0.025, Figure S1). This is also the case for Anxious-depression: Cohen’s f^2=-0.030 when other 2 factors from the solution are included in the model (Figure 3A), Cohen’s f^2=-0.022 when factors are in separate models (Figure S1). This demonstrates the importance of including factors from the same solution in a single regression model, to control for factors with opposing effects on metacognitive bias (e.g., ‘Anxious-Depression’ vs. ‘Compulsivity and Intrusive Thought’).

Scores on the 2nd factor from this same 2-factor solution were also highly correlated with ‘Compulsivity and Intrusive Thought’, $r(207)=.93$, $p<0.001$ (Figure S2A), and loadings were correlated at $r(207)=0.84$, $p<0.001$, with the main difference in loadings also being attributed to social-anxiety items (Figure S2B), which in a 3-factor solution forms its own factor termed ‘Social Withdrawal’ in prior work (Gillan et al., 2016).

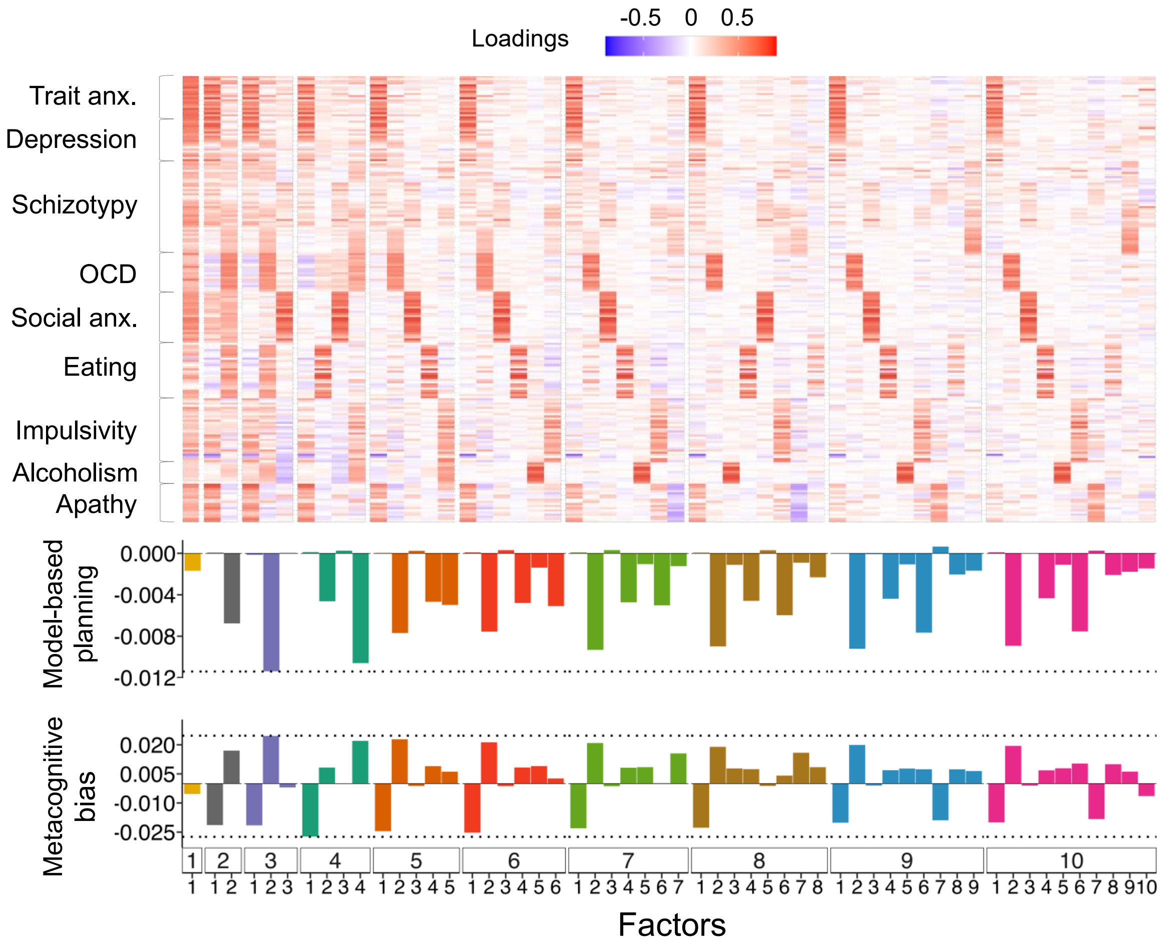

Figure S1. Re-analyses of variation in the number of factors retained, without controlling for other factors from that model. Fifty-five factors were generated from solutions retaining 1-factor, 2-factors, …, 10-factors. Heatmap indicates the loading of individual items onto each resulting factor (top panel). Weighted effect sizes from 10 linear regression analysis predicting model-based planning, averaged across 5 datasets (N=4990) (middle panel) and metacognition in 4 datasets (N=2575) (bottom panel), when factors are included in separate regression models.

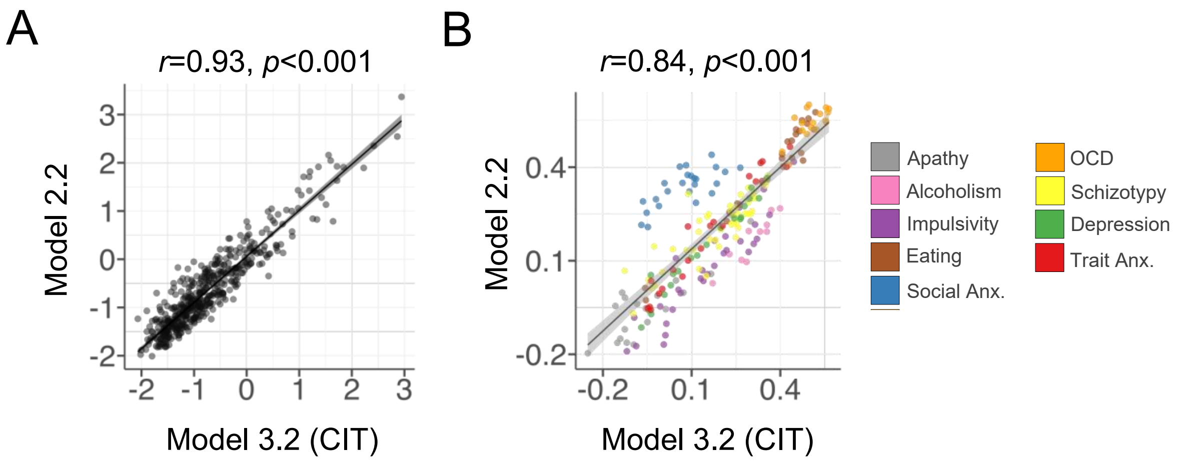

Figure S2. Re-analyses of variation in the number of factors retained, without controlling for other factors from that model. CIT=Compulsivity and Intrusive Thought. **(A)** Correlation between scores on the 2nd factor in a 3-factor solution (model 3.2 i.e, 'Compulsivity and Intrusive Thought') and the 2nd factor from two factor solution (model 2.2) in the discovery dataset. **(B)** Correlation between factor loadings of Compulsivity and Intrusive Thought and model 2.2 in the discovery dataset.

Variation in the selection of clinical symptoms

For the association with deficits in model-based planning, the top performing factor and ‘Compulsivity and intrusive thought’ both had highest loadings for OCD items and the lowest loadings for apathy items, with the top factor being generated from a set of questionnaires that omitted the schizotypy and alcohol addiction scales (Figure S3A). Examining loadings for the top performing factor that predicted overconfidence omitted the trait anxiety and eating disorder questionnaires were omitted (Figure S3B). While the top performing factor for associations with underconfidence also omitted the eating disorder questionnaire, this factor had the highest loadings for the trait anxiety items (Figure S3C).

Visualising the item-level loadings across the top 1500 dimensions associated with deficits in model-based planning, items related to obsessive-compulsive disorder appears to have relatively stronger positive loadings across dimensions (Figure S4A). This was confirmed when averaging item-level loadings across the top 100 dimensions (Figure S5A). Items related to obsessive compulsive disorder (M=0.51, SD=0.01), followed by eating disorders (M=0.20, SD=0.07), had the highest average questionnaire-level loadings, while apathy items had the lowest average loading (M=-0.01, SD=0.03) across the top 100 dimensions associated with deficits in model-based planning (Figure S5D). For associations with overconfidence, a heatmap of the top 1500 dimensions illustrated the relative importance of items related to obsessive-compulsive disorder, impulsivity and schizotypy (Figure S4B). Similar to the top dimensions associated with deficits in model-based planning, items from the obsessive compulsive disorder questionnaire contributed most predominantly to the top 100 dimensions (Figure S5B) and had the highest questionnaire-level average loadings (M=0.36, SD=0.02) (Figure S5D). Additionally, items related to impulsivity (M=0.22, SD=0.04) and schizotypy (M=0.20, SD = 0.03) had relatively higher average loadings on the top 100 dimensions that explained individual differences in overconfidence (Figure S5B-D).

When examining negative associations with confidence bias, trait anxiety items had strong positive loadings across the top 1500 performing dimensions (Figure S4C). The relative importance of trait anxiety items was confirmed by averaging loadings across the top 100 dimensions, at the item-level (Figure S5C) and questionnaire-level ((M=0.59, SD=0.02) (Figure S5D). In contrast to trait anxiety, the depression questionnaire was completely omitted from the top 100 dimensions (Figures S4C & S5C), indicating that removal of the depression questionnaire generates dimensions sensitive to individual differences in underconfidence. The relative importance of trait anxiety was specific to underconfidence, as trait anxiety had lower loadings across the top 100 dimensions predicting model-based planning (M=0.06, SD=0.05) and overconfidence (M=0.06, SD=0.05). While obsessive compulsive disorder items had the highest loadings across the top 100 dimensions for overconfidence (M=0.36, SD=0.02) (Figures S5B and S5D), obsessive compulsive disorder items did not load relatively highly across dimensions for underconfidence (M=0.02, SD=0.03) (Figures S5C-D). This suggested that over- and under-confidence have differential associations with psychopathology (i.e., overconfidence with OCD vs. underconfidence with trait anxiety), rather than merely representing effects with opposing directionality on the same clinical phenomena.

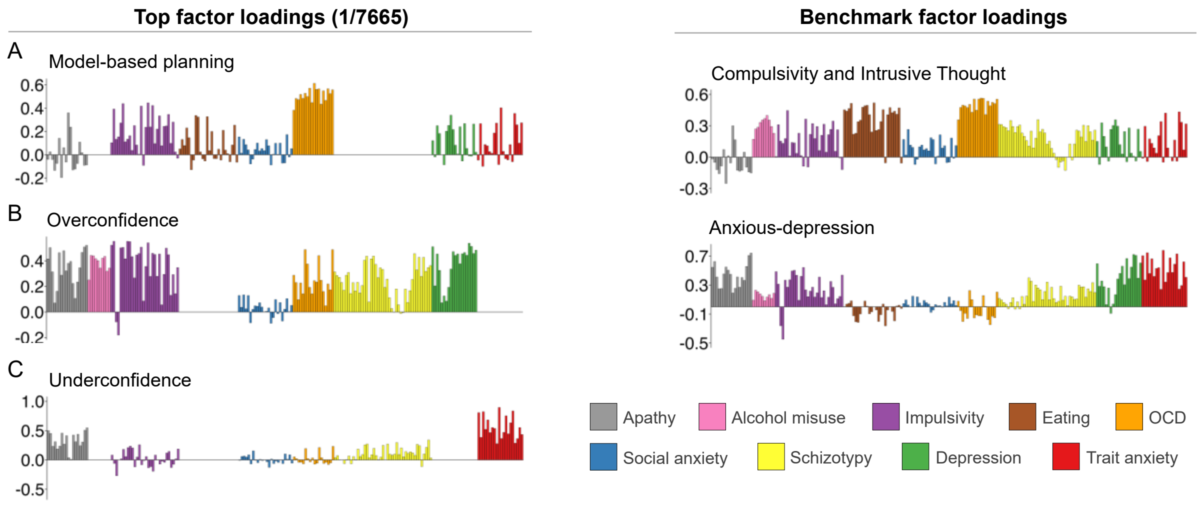

Figure S3. Top (1/7665) performing dimensions from varying the selection of clinical symptoms. OCD = obsessive compulsive disorder. The left side of the panel includes loadings for the 209 questionnaire items across the top performing factors with largest effect sizes for the associations with **(A)** model-based planning, **(B)** overconfidence, and **(C)** underconfidence (left-side of panel). On the right-side are loadings for the 209 items on the benchmark dimensions ('Compulsivity and Intrusive Thought' and 'Anxious-depression').

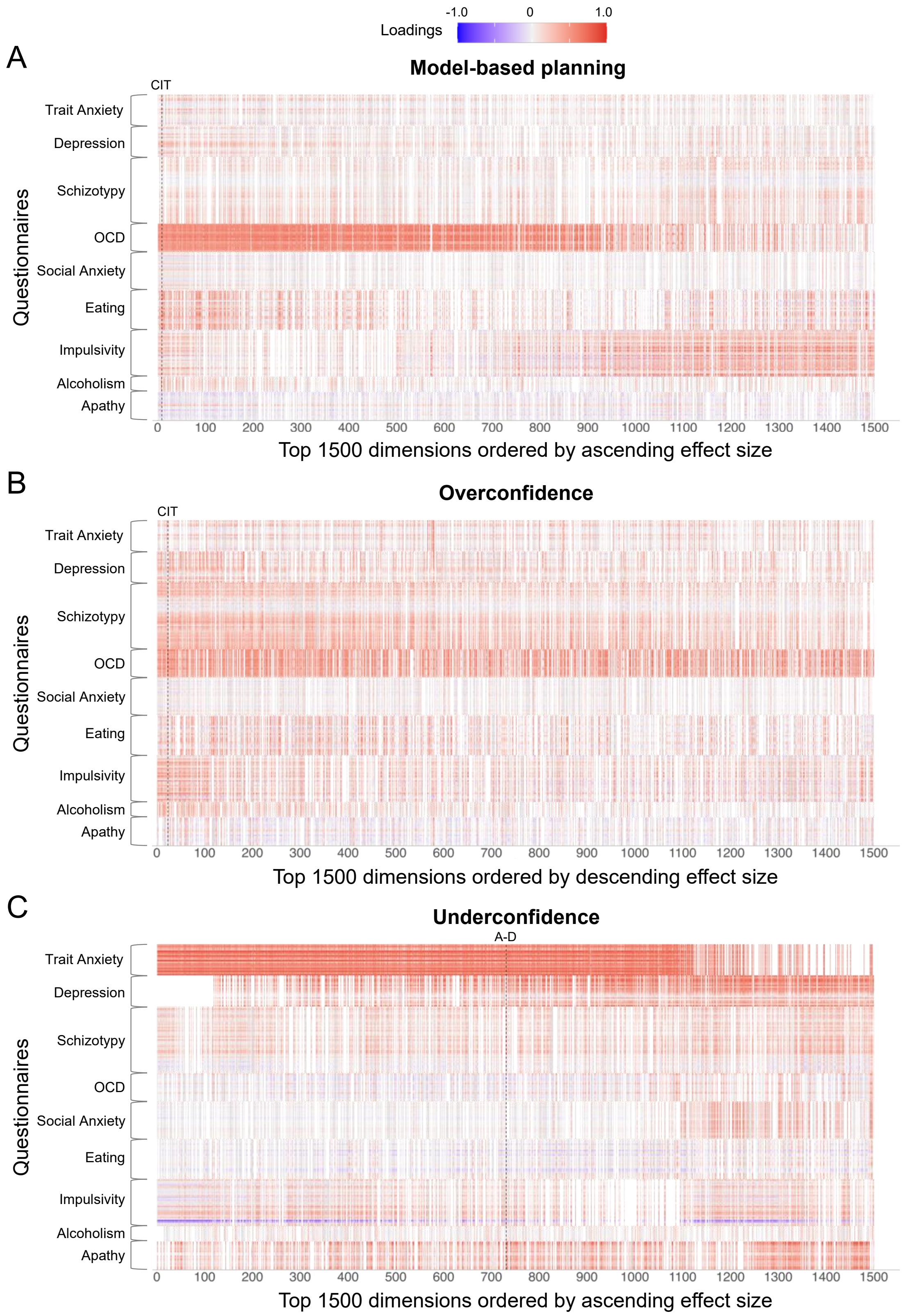

Figure S4. Heatmaps of top 1500/7665 performing dimensions from varying the selection of clinical symptoms. CIT=Compulsivity and Intrusive Thought. A-D=Anxious-depression, OCD= Obsessive compulsive disorder. Heatmaps for top 1500 dimensions with largest weighted average effect sizes for associations with **(A)** model-based planning, **(B)** overconfidence (positive mean confidence), and **(C)** underconfidence (negative mean confidence).

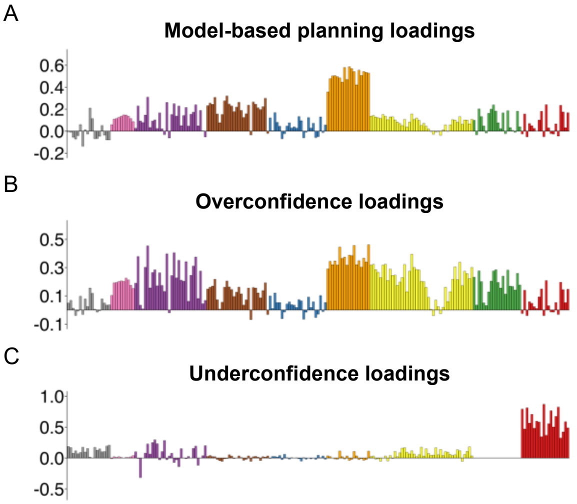

D

Top 100 dimensions: Questionnaire-level average loadings

Questionnaire	Model-based planning	Over-confidence	Under-confidence
Apathy	-0.02 (0.03)	0.04 (0.03)	0.12 (0.07)
Alcoholism	0.12 (0.03)	0.19 (0.03)	0.01 (0.01)
Impulsivity	0.12 (0.05)	0.22 (0.04)	0.04 (0.02)
Eating	0.20 (0.07)	0.12 (0.05)	0.004 (0.02)
Social Anxiety	0.04 (0.02)	0.03 (0.02)	0.004 (0.02)
OCD	**0.51 (0.01)**	**0.36 (0.02)**	0.02 (0.03)
Schizotypy	0.08 (0.05)	0.20 (0.03)	0.06 (0.04)
Depression	0.10 (0.04)	0.18 (0.06)	
Trait Anxiety	0.06 (0.05)	0.06 (0.06)	**0.59 (0.02)**

Figure S5. Average across top 100/7665 performing dimensions from varying the selection of clinical symptoms. OCD = Obsessive compulsive disorder. Average loadings for the 209 questionnaire items across the top 100 dimensions with largest effect sies for the associations with **(A)** model-based planning, **(B)** overconfidence, and **(C)** underconfidence. **(D)** The average questionnaire-level loadings across the top 100 dimensions associated with cognitive outcomes. Questionnaire colours in (D) correspond to item colours in (A-C) (e.g., grey = apathy item-/questionnaire-level loadings).

Higher- versus first-order factor rotation

The within-solution correlations between factor scores verified the oblique and orthogonal nature across rotation types. The oblique rotation 'oblimin' produces correlations between factors 1,2 and 3 ranging from 0.28-0.43, compared to the orthogonal rotation 'varimax', which ranged from r=-.06 to .08 (Figure S6). Applying a bifactor rotation produced factor structures that were internally orthogonal, with correlations between factors 1,2, 3 and the hierarchical general 'G' factor all at r=0 (Figure S6).

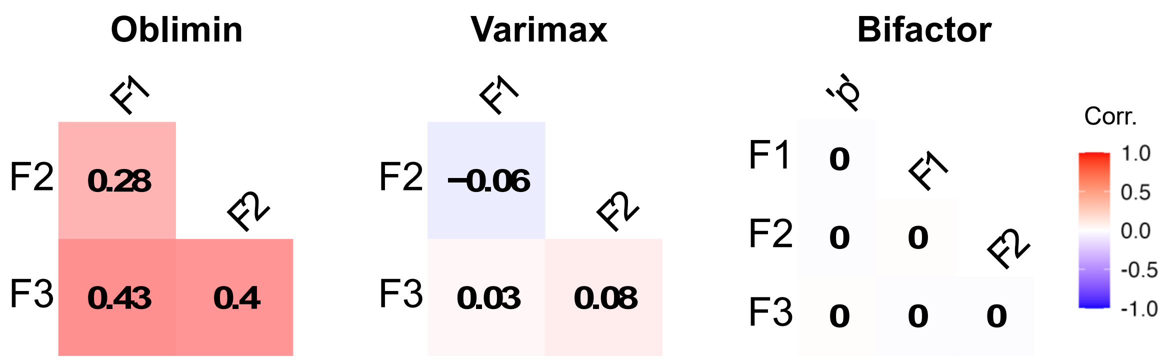

Figure S6. Higher- versus first-order factor rotation: The within-solution correlations between factor scores. F1= factor 1, F2 = factor 2, F3 = factor 3, 'p' = general factor. The within-solution correlations indicated strong positive correlations within the oblimin (oblique) solution, and very weak/no correlation between factors within the orthogonal solutions 'varimax' and the bifactor model.

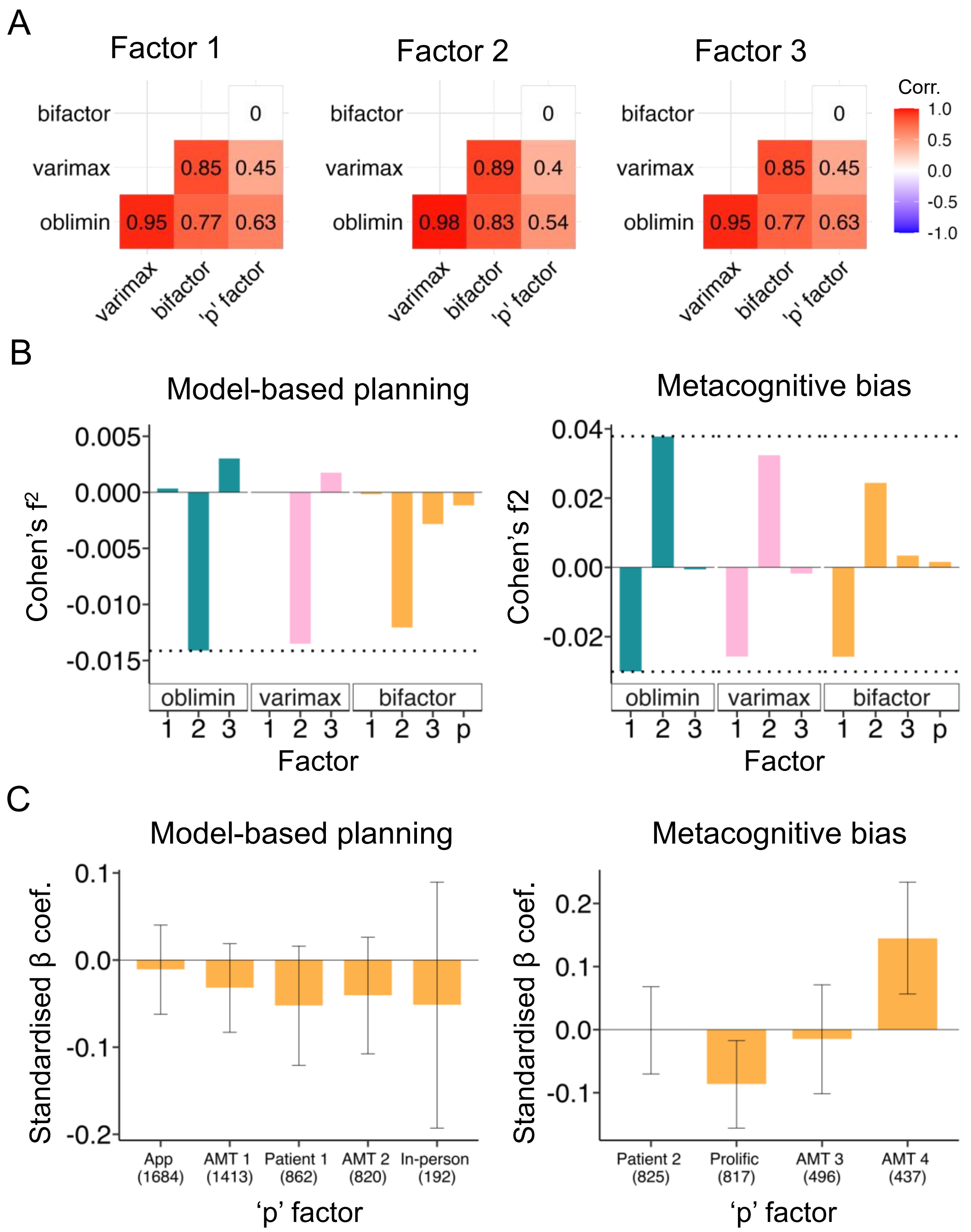

Figure S7. Higher- versus first-order factor rotation. AMT = Amazon's Mechanical Turk. p = general factor extracted from bifactor model. (A) Correlation of scores on factor 1, 2 and 3 from the 3 derivations – oblique, orthogonal and bifactor models in the discovery dataset (N=1413). Each correlation matrix also includes the correlation to the bifactor models general ('p') factor. (B) Weighted effect size for the association between derived factors and model-based planning and metacognitive bias. An oblique solution produces the winning model in all cases. (C) Individual results from component datasets for the 'p' factor reveals no consistent pattern of association with the cognitive constructs in question.

Partial Least Squares Regression

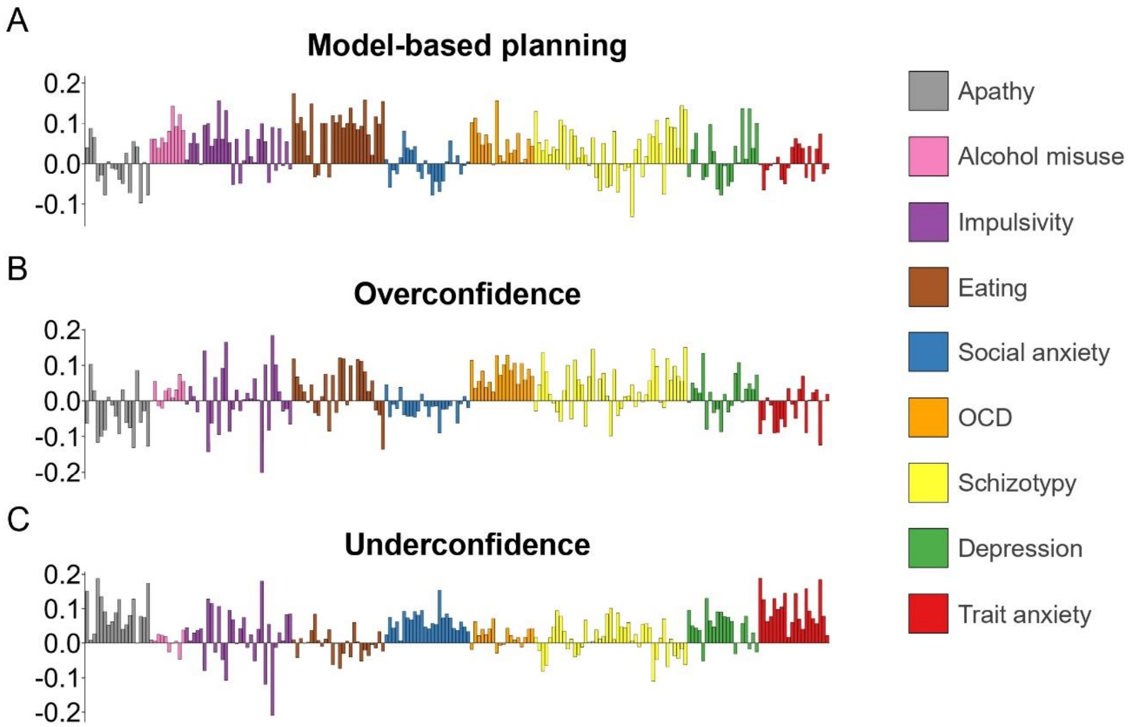

Figure S8. Weights of items across components generated with Partial Least Squares Regression. OCD = Obsessive compulsive disorder. The 209 questionnaire item weights for the components generated with partial least squares regression to predict individual differences in **(A)** model-based planning, **(B)** overconfidence, and **(C)** underconfidence.

Table S1. Individual questionnaire items and weights for the Partial Least Squares Regression solutions, Anxious-depression and Compulsivity and Intrusive Thought.

AES = Apathy (Apathy Evaluation Scale), AUD = Alcohol misuse (Alcohol Use Disorders Identification Test), BIS = Impulsivity (Barratt Impulsiveness Scale 11), EAT = Eating (Eating Attitudes Test), LSA = Social anxiety (Liebowitz Social Anxiety Scale), OCI = OCD (Obsessive-Compulsive Inventory-Revised, OCI-R), SCZ = Schizotypy (Short Scales for Measuring Schizotypy), SDS = Depression (Zung Self-Rating Depression Scale), STA = Trait anxiety (State Trait Anxiety Inventory), pls_mbi = weights for the PLS factor predicting model-based planning (negative association), pls_overconf = weights for the PLS factor predicting (over)confidence (positive association), pls_underconf = weights for the PLS factor predicting (under)confidence (negative association), AD = Anxious-depression, CIT = Compulsivity and Intrusive Thought.

Questionnaire	Item	PLS-MBI	PLS-overconf	CIT	PLS-underconf	AD
AES	AES_1	0.04	-0.06	-0.01	0.15	0.02
AES	AES_2	-0.05	0.03	-0.01	0.04	0.03
AES	AES_3	0.03	-0.06	-0.01	0.05	0.02
AES	AES_4	-0.07	-0.07	-0.01	0.08	0.01
AES	AES_5	0.06	-0.13	-0.01	0.13	0.01
AES	AES_6	0.04	0.09	0.00	-0.00	0.02
AES	AES_7	-0.10	-0.06	-0.02	0.08	0.03
AES	AES_8	0.00	-0.03	-0.00	0.07	0.02
AES	AES_9	-0.08	-0.13	-0.01	0.17	0.02
AES	AES_10	0.09	0.10	0.02	0.01	0.01
AES	AES_11	0.06	0.03	0.01	0.03	0.01
AES	AES_12	-0.04	-0.12	-0.01	0.19	0.02
AES	AES_13	-0.03	-0.10	-0.01	0.13	0.01
AES	AES_14	-0.08	-0.08	-0.01	0.09	0.02
AES	AES_15	0.01	0.01	0.00	0.05	0.01
AES	AES_16	-0.01	-0.01	-0.01	0.06	0.02
AES	AES_17	-0.01	-0.04	-0.02	0.09	0.04
AES	AES_18	-0.04	-0.09	-0.02	0.13	0.05
AUD	AUDIT_1	0.06	-0.00	0.01	0.01	0.00
AUD	AUDIT_2	0.04	-0.01	0.01	0.02	0.01
AUD	AUDIT_3	0.06	-0.02	0.02	0.02	0.01
AUD	AUDIT_4	0.05	0.03	0.02	0.02	0.01
AUD	AUDIT_5	0.08	0.04	0.02	-0.03	0.00
AUD	AUDIT_6	0.14	0.01	0.02	-0.00	0.00
AUD	AUDIT_7	0.09	0.03	0.02	0.01	0.00
AUD	AUDIT_8	0.12	0.07	0.02	-0.05	0.01
AUD	AUDIT_9	0.08	0.06	0.02	0.04	0.00
AUD	AUDIT_10	0.06	0.05	0.01	0.01	0.01
BIS	BIS_1	0.01	-0.01	-0.00	0.05	0.02
BIS	BIS_2	0.13	0.16	0.02	-0.11	0.01
BIS	BIS_3	0.05	0.10	0.01	-0.12	-0.01
BIS	BIS_4	0.09	0.18	0.01	-0.21	-0.02
BIS	BIS_5	-0.02	0.10	0.01	-0.01	0.01
BIS	BIS_6	0.09	0.03	0.03	0.03	0.01
BIS	BIS_7	-0.00	-0.03	-0.00	0.01	0.01
BIS	BIS_8	0.05	-0.02	0.01	0.08	0.02
BIS	BIS_9	-0.01	-0.06	0.01	0.09	0.02
BIS	BIS_10	0.05	0.04	-0.00	0.01	0.02
BIS	BIS_11	0.08	0.01	0.01	0.03	0.00
BIS	BIS_12	0.05	-0.03	-0.00	0.04	0.02
BIS	BIS_13	-0.00	-0.00	-0.01	0.04	0.02
BIS	BIS_14	0.09	0.14	0.02	-0.08	0.01
BIS	BIS_15	0.10	-0.14	-0.01	0.13	0.00
BIS	BIS_16	0.04	-0.06	0.01	0.12	0.01
BIS	BIS_17	0.06	0.07	0.02	-0.03	0.01
BIS	BIS_18	0.16	-0.09	0.01	0.11	0.00
BIS	BIS_19	0.06	0.09	0.02	-0.05	0.01
BIS	BIS_20	0.05	-0.09	0.00	0.10	0.02
BIS	BIS_21	-0.05	-0.02	0.01	0.07	0.00
BIS	BIS_22	0.06	0.03	0.02	-0.00	0.01
BIS	BIS_23	-0.05	-0.03	0.00	0.04	0.00
BIS	BIS_24	0.01	0.00	0.01	0.07	0.00
BIS	BIS_25	0.08	0.02	0.01	-0.01	0.01
BIS	BIS_26	0.02	0.01	0.02	0.02	0.01
BIS	BIS_27	0.00	0.06	0.00	-0.05	0.00
BIS	BIS_28	0.06	0.00	0.01	0.04	0.00
BIS	BIS_29	0.10	-0.20	-0.00	0.18	0.00
BIS	BIS_30	-0.05	-0.08	-0.01	0.06	0.02
EAT	EAT_1	0.17	0.12	0.03	0.01	-0.00
EAT	EAT_2	0.12	0.07	0.03	-0.06	-0.00
EAT	EAT_3	0.09	0.11	0.03	-0.05	-0.00
EAT	EAT_4	0.16	0.08	0.03	-0.02	-0.00
EAT	EAT_5	0.07	0.03	0.01	-0.04	-0.01
EAT	EAT_6	0.02	0.06	0.01	-0.00	-0.01
EAT	EAT_7	0.12	-0.03	0.02	-0.01	-0.01
EAT	EAT_8	0.10	-0.04	0.02	0.03	-0.01
EAT	EAT_9	0.15	-0.14	0.03	-0.02	-0.00
EAT	EAT_10	0.10	0.07	0.03	-0.04	-0.00
EAT	EAT_11	0.12	0.04	0.03	0.01	-0.00
EAT	EAT_12	0.08	0.03	0.02	-0.00	-0.01
EAT	EAT_13	0.02	-0.00	0.01	0.00	-0.00
EAT	EAT_14	0.15	0.04	0.04	0.04	-0.01
EAT	EAT_15	-0.03	-0.03	0.01	0.08	-0.00
EAT	EAT_16	-0.03	-0.04	0.01	0.01	-0.01
EAT	EAT_17	0.10	0.01	0.02	-0.01	-0.01
EAT	EAT_18	0.10	0.08	0.03	0.01	-0.00
EAT	EAT_19	-0.03	-0.08	-0.00	0.04	-0.01
EAT	EAT_20	0.10	-0.03	0.02	-0.00	-0.00
EAT	EAT_21	0.12	0.12	0.03	-0.07	-0.00
EAT	EAT_22	0.09	0.12	0.03	-0.03	-0.01
EAT	EAT_23	0.10	-0.01	0.03	0.01	-0.01
EAT	EAT_24	0.14	0.10	0.02	-0.04	-0.00
EAT	EAT_25	0.10	0.00	0.03	0.06	-0.00
EAT	EAT_26	0.08	0.12	-0.00	0.00	-0.00
LSA	LSAS_1	0.01	0.04	0.01	0.02	-0.00
LSA	LSAS_2	0.01	-0.01	-0.01	0.05	0.00
LSA	LSAS_3	0.01	-0.02	0.01	0.09	-0.00
LSA	LSAS_4	0.06	-0.01	0.00	0.07	-0.00
LSA	LSAS_5	0.00	-0.00	0.00	0.04	0.00
LSA	LSAS_6	0.02	-0.06	-0.01	0.04	-0.00
LSA	LSAS_7	-0.03	-0.01	-0.01	0.06	0.00
LSA	LSAS_8	-0.00	0.01	0.00	0.05	-0.00
LSA	LSAS_9	0.00	-0.02	0.00	0.03	-0.00
LSA	LSAS_10	-0.06	-0.04	-0.00	0.08	-0.00
LSA	LSAS_11	-0.02	-0.01	0.00	0.04	-0.00
LSA	LSAS_12	-0.03	-0.02	-0.00	0.06	-0.01
LSA	LSAS_13	0.02	0.04	0.01	0.01	-0.01
LSA	LSAS_14	0.08	-0.04	0.01	0.09	-0.01
LSA	LSAS_15	0.04	-0.04	-0.02	0.07	-0.00
LSA	LSAS_16	0.03	-0.04	-0.01	0.07	-0.01
LSA	LSAS_17	0.04	-0.05	0.01	0.09	-0.00
LSA	LSAS_18	-0.02	-0.03	0.00	0.08	-0.01
LSA	LSAS_19	-0.04	0.02	0.00	0.10	-0.00
LSA	LSAS_20	-0.03	-0.04	-0.01	0.04	-0.00
LSA	LSAS_21	-0.08	-0.02	-0.01	0.06	0.00
LSA	LSAS_22	-0.04	-0.01	0.01	0.05	-0.00
LSA	LSAS_23	-0.07	-0.09	-0.01	0.15	0.00
LSA	LSAS_24	-0.04	-0.02	0.00	0.07	-0.00
OCI	OCI_1	0.10	0.11	0.02	-0.02	0.00
OCI	OCI_2	0.06	0.13	0.03	0.04	-0.01
OCI	OCI_3	0.03	0.09	0.03	0.02	-0.01
OCI	OCI_4	0.03	0.11	0.03	0.01	-0.01
OCI	OCI_5	0.07	0.05	0.03	0.02	-0.01
OCI	OCI_6	0.00	0.11	0.04	-0.01	0.01
OCI	OCI_7	0.01	0.06	0.03	0.02	0.00
OCI	OCI_8	0.06	0.09	0.04	0.04	-0.01
OCI	OCI_9	0.04	0.07	0.03	0.04	-0.01
OCI	OCI_10	0.11	0.03	0.04	0.06	-0.01
OCI	OCI_11	0.08	0.06	0.04	0.02	-0.01
OCI	OCI_12	0.05	0.08	0.05	0.04	0.00
OCI	OCI_13	0.07	0.04	0.02	0.02	-0.00
OCI	OCI_14	0.05	0.05	0.04	0.04	-0.01
OCI	OCI_15	-0.00	0.02	0.03	0.07	-0.02
OCI	OCI_16	0.16	0.13	0.03	-0.03	-0.01
OCI	OCI_17	0.02	0.07	0.03	-0.01	-0.01
OCI	OCI_18	0.01	0.10	0.05	-0.00	0.01
SCZ	SCZ_1	0.13	-0.03	0.02	0.02	0.00
SCZ	SCZ_2	0.07	0.11	0.02	-0.04	0.00
SCZ	SCZ_3	0.08	0.09	0.02	0.01	0.00
SCZ	SCZ_4	0.07	0.14	0.01	-0.11	-0.00
SCZ	SCZ_5	0.09	0.06	0.01	0.01	-0.00
SCZ	SCZ_6	0.09	0.10	0.02	-0.04	-0.00
SCZ	SCZ_7	0.04	0.07	0.02	-0.04	0.00
SCZ	SCZ_8	0.14	0.06	0.01	-0.02	-0.00
SCZ	SCZ_9	0.13	0.15	0.02	-0.06	0.00
SCZ	SCZ_10	0.05	0.04	0.02	-0.02	-0.00
SCZ	SCZ_11	0.03	0.13	0.01	-0.08	0.00
SCZ	SCZ_12	0.06	0.08	0.01	-0.06	-0.00
SCZ	SCZ_13	0.02	0.02	0.01	0.02	0.00
SCZ	SCZ_14	0.04	-0.01	0.00	0.05	0.02
SCZ	SCZ_15	0.03	-0.03	0.01	0.09	0.01
SCZ	SCZ_16	0.11	-0.06	0.00	0.08	-0.00
SCZ	SCZ_17	0.09	0.03	0.01	0.03	0.01
SCZ	SCZ_18	-0.01	0.10	0.02	-0.02	0.01
SCZ	SCZ_19	0.08	-0.05	0.01	0.01	0.01
SCZ	SCZ_20	0.01	0.06	0.01	-0.03	0.00
SCZ	SCZ_21	0.04	0.05	0.01	-0.01	0.01
SCZ	SCZ_22	0.02	-0.07	0.00	0.07	0.01
SCZ	SCZ_23	-0.00	0.07	0.01	0.00	0.00
SCZ	SCZ_24	0.06	0.14	0.00	0.00	0.01
SCZ	SCZ_25	-0.04	0.00	0.00	0.04	0.00
SCZ	SCZ_26	-0.07	0.06	-0.00	0.01	0.00
SCZ	SCZ_27	0.05	0.08	-0.01	0.02	0.00
SCZ	SCZ_28	-0.06	0.01	-0.01	0.08	0.01
SCZ	SCZ_29	-0.06	-0.10	-0.00	0.10	0.00
SCZ	SCZ_30	-0.07	-0.04	-0.01	0.09	0.00
SCZ	SCZ_31	-0.00	-0.02	-0.00	0.05	-0.00
SCZ	SCZ_32	-0.02	0.01	0.00	0.05	0.00
SCZ	SCZ_33	-0.01	0.01	-0.00	0.06	0.00
SCZ	SCZ_34	-0.13	0.04	0.01	0.01	0.00
SCZ	SCZ_35	0.03	-0.02	0.01	0.02	0.00
SCZ	SCZ_36	0.06	0.02	0.01	-0.01	0.01
SCZ	SCZ_37	-0.03	-0.00	0.01	0.05	0.01
SCZ	SCZ_38	0.02	0.02	0.02	0.06	0.01
SCZ	SCZ_39	0.07	0.06	0.01	0.05	0.00
SCZ	SCZ_40	0.11	0.10	0.02	-0.05	0.01
SCZ	SCZ_41	0.05	-0.00	0.01	0.01	0.01
SCZ	SCZ_42	-0.08	-0.02	0.01	0.07	0.01
SCZ	SCZ_43	0.11	0.12	0.01	-0.07	0.01
SDS	SDS_1	-0.03	-0.00	0.01	0.10	0.03
SDS	SDS_2	-0.06	-0.03	-0.00	0.08	0.01
SDS	SDS_3	0.04	0.08	0.02	-0.03	0.01
SDS	SDS_4	0.00	0.11	0.01	-0.00	0.01
SDS	SDS_5	0.14	-0.00	0.00	0.06	0.00
SDS	SDS_6	0.01	0.03	-0.00	0.08	0.01
SDS	SDS_7	0.14	0.05	0.01	-0.02	-0.00
SDS	SDS_8	0.04	0.03	0.02	0.03	-0.00
SDS	SDS_9	0.10	0.07	0.02	-0.03	0.00
SDS	SDS_10	0.03	0.05	0.02	0.05	0.02
SDS	SDS_11	0.08	0.03	0.00	0.05	0.02
SDS	SDS_12	0.00	0.02	-0.00	0.04	0.03
SDS	SDS_13	-0.04	0.13	0.01	-0.05	0.01
SDS	SDS_14	-0.03	-0.08	-0.01	0.13	0.04
SDS	SDS_15	0.10	0.02	0.02	0.07	0.02
SDS	SDS_16	0.03	-0.03	-0.00	0.05	0.03
SDS	SDS_17	-0.06	-0.02	-0.01	0.09	0.05
SDS	SDS_18	-0.08	-0.09	-0.01	0.09	0.04
SDS	SDS_19	-0.01	0.02	0.01	0.08	0.02
SDS	SDS_20	-0.05	-0.01	-0.00	0.06	0.03
STA	STAI_1	-0.00	-0.09	-0.01	0.19	0.05
STA	STAI_2	0.05	0.03	0.02	0.07	0.02
STA	STAI_3	-0.04	-0.09	-0.01	0.16	0.05
STA	STAI_4	0.04	0.00	0.01	0.09	0.02
STA	STAI_5	-0.04	0.02	0.01	0.07	0.05
STA	STAI_6	0.04	0.03	0.00	0.06	0.02
STA	STAI_7	0.07	-0.12	-0.00	0.18	0.02
STA	STAI_8	-0.03	-0.00	0.01	0.08	0.03
STA	STAI_9	-0.01	0.02	0.02	0.02	0.01
STA	STAI_10	-0.07	-0.05	-0.02	0.13	0.07
STA	STAI_11	-0.02	0.01	0.03	0.06	0.01
STA	STAI_12	-0.00	-0.01	0.00	0.08	0.03
STA	STAI_13	0.00	-0.09	-0.01	0.13	0.04
STA	STAI_14	0.02	-0.09	-0.01	0.10	0.02
STA	STAI_15	-0.04	-0.05	0.01	0.11	0.04
STA	STAI_16	-0.05	-0.07	-0.01	0.14	0.05
STA	STAI_17	-0.01	0.03	0.03	0.02	0.01
STA	STAI_18	0.04	-0.01	0.02	0.06	0.01
STA	STAI_19	0.06	-0.05	-0.00	0.14	0.03
STA	STAI_20	0.04	0.07	0.02	0.04	0.02
